# Metagenomic and metaproteomic analyses of a corn stover-adapted microbial consortium EMSD5 reveal its taxonomic and enzymatic basis for degrading lignocellulose

**DOI:** 10.1186/s13068-016-0658-z

**Published:** 2016-11-09

**Authors:** Ning Zhu, Jinshui Yang, Lei Ji, Jiawen Liu, Yi Yang, Hongli Yuan

**Affiliations:** 1 State Key Laboratory of Agrobiotechnology, College of Biological Sciences, Beijing, China; 2 National Energy R & D Center for Non-food Biomass, China Agricultural University, Beijing, 100193 China

**Keywords:** Plant biomass, Microbial consortium, Corn stover, Metagenomics, Metaproteomics, *Firmicutes*, Synergism, Hemicellulase

## Abstract

**Background:**

Microbial consortia represent promising candidates for aiding in the development of plant biomass conversion strategies for biofuel production. However, the interaction between different community members and the dynamics of enzyme complements during the lignocellulose deconstruction process remain poorly understood. We present here a comprehensive study on the community structure and enzyme systems of a lignocellulolytic microbial consortium EMSD5 during growth on corn stover, using metagenome sequencing in combination with quantitative metaproteomics.

**Results:**

The taxonomic affiliation of the metagenomic data showed that EMSD5 was primarily composed of members from the phyla *Proteobacteria*, *Firmicutes* and *Bacteroidetes*. The carbohydrate-active enzyme (CAZyme) annotation revealed that representatives of *Firmicutes* encoded a broad array of enzymes responsible for hemicellulose and cellulose deconstruction. Extracellular metaproteome analysis further pinpointed the specific role and synergistic interaction of *Firmicutes* populations in plant polysaccharide breakdown. In particular, a wide range of xylan degradation-related enzymes, including xylanases, β-xylosidases, α-l-arabinofuranosidases, α-glucuronidases and acetyl xylan esterases, were secreted by diverse members from *Firmicutes* during growth on corn stover. Using label-free quantitative proteomics, we identified the differential secretion pattern of a core subset of enzymes, including xylanases and cellulases with multiple carbohydrate-binding modules (CBMs). In addition, analysis of the coordinate expression patterns indicated that transport proteins and hypothetical proteins may play a role in bacteria processing lignocellulose. Moreover, enzyme preparation from EMSD5 demonstrated synergistic activities in the hydrolysis of pretreated corn stover by commercial cellulases from *Trichoderma reesei*.

**Conclusions:**

These results demonstrate that the corn stover-adapted microbial consortium EMSD5 harbors a variety of lignocellulolytic anaerobic bacteria and degradative enzymes, especially those implicated in hemicellulose decomposition. The data in this study highlight the pivotal role and cooperative relationship of *Firmicutes* members in the biodegradation of plant lignocellulose by EMSD5. The differential expression patterns of enzymes reveal the strategy of sequential lignocellulose deconstruction by EMSD5. Our findings provide insights into the mechanism by which consortium members orchestrate their array of enzymes to degrade complex lignocellulosic biomass.

**Electronic supplementary material:**

The online version of this article (doi:10.1186/s13068-016-0658-z) contains supplementary material, which is available to authorized users.

## Background

Plant biomass is considered as an abundant renewable resource that can be transformed into fermentable sugars for biofuel production. In nature, plant biomass degradation is mediated mostly by lignocellulolytic microorganisms, which have evolved distinct enzymatic systems for complex polysaccharide degradation. Aerobic fungi and most bacteria, such as *Trichoderma reesei* and *Thermobifida fusca*, secrete a broad range of free enzymes with different substrate specificities to synergistically degrade plant polysaccharides [1, 2]. In contrast, a few anaerobic bacteria represented by *Clostridium thermocellum* integrate various cellulases and xylanases subunits into large multi-enzyme complexes known as cellulosomes [3, 4]. An intermediate strategy involved the action of multifunctional hydrolases with multiple glycoside hydrolase (GH) and carbohydrate-binding module (CBM) domains, as exemplified by the multi-modular CelA from the extreme thermophile *Caldicellulosiruptor bescii* [5]. Our current approaches of developing more efficient enzyme cocktails and improving biomass conversion rely largely on expanding our knowledge on the microbial degradation and utilization of plant biomass.

In natural ecosystems, biodegradation of lignocellulosic biomass is usually accomplished by intricate consortia of diverse microorganisms rather than individuals. Numerous studies have revealed significant diversity in lignocellulolytic microbial communities from various growth environments, including compost [6], forest soil [7], poplar wood chips [8], sugarcane bagasse [9], cow rumen [10], termite hindgut [11] and biogas reactor [12]. It is presumed that taxonomically different members within the microbial communities work in cooperation to break down the plant cell wall polysaccharides. Therefore, microbial consortia provide an excellent paradigm for studying the interplay between distinct microorganisms as well as their lignocellulolytic enzyme systems, which can be of benefit in the design of enzyme cocktails based on enzymatic synergy. Also, fascinating from an industrial perspective is the proposal of using enzyme mixtures produced by microbial consortia to saccharify pretreated biomass, as multispecies consortia tend to have more balanced enzyme complements than single strains. These enzymes can also be exploited for their environmental bioremediation potential [13]. Moreover, through oriented enrichment, the enzyme repertoire of the resulting consortia can be tailored to deconstruct particular lignocellulosic feedstock under industrially relevant conditions [14, 15].

The application of metagenomic approaches has been proven to be very useful in unveiling the biodegradative potential of microbial consortia and recovering genes encoding novel enzymes with improved properties, as evidenced in a recent discovery of highly halotolerant and ionic liquid-resistant cellulases using a metagenomics-guided strategy [16]. Likewise, a targeted metagenomic approach has been used to facilitate the identification of four hemicellulases with moderate thermal stability and broad pH optima from a switchgrass-adapted compost microbial community [17]. In addition, analysis of the taxonomic affiliation of genes related to lignocellulose degradation enables us to correlate specific functions with abundant microbial groups and to characterize their potential synergistic action [18]. Our capability to explore the microbial enzymology of plant biomass deconstruction is also being augmented by recent advances in metaproteomic methods. While metagenome studies inform about the genetic backgrounds of plant biomass degradation by microbial consortia, extracellular metaproteomics provides a more focused picture of the lignocellulolytic apparatus secreted by a microbial consortium under defined conditions. Comparisons of the taxonomic structures and secreted proteins of microbial consortia responsive to substrates with distinct complexity could help to understand their unique enzyme systems [19]. Additionally, such comparative analyses offer the opportunity to explore lignocellulose-depolymerizing mechanisms of microbial consortia and to evaluate their potential applications in industrial biomass conversion.

Although previous metaproteome studies have revealed much about lignocellulose degradation in microbial consortia, they are limited to the analyses of proteins at a single time point [19, 20]. We hypothesize that in the process of plant biomass deconstruction, variations occur in the microbial consortia with respect to the taxonomic composition, degradative enzyme activities and protein expression levels. Thus, an investigation of the temporal dynamics of extracellular protein profiles by quantitative proteomics will help to distinguish the differential expression of enzymes involved in plant biomass processing, thereby unraveling the lignocellulose-degrading strategies employed by the consortia.

Corn stover is a readily available agricultural waste of high yields globally that can serve as the lignocellulosic feedstock for the production of second-generation bioethanol [21]. In our previous work, a microbial consortium EMSD5 was enriched from compost habitats and adapted to grow on unpretreated corn stover [22]. The extracellular fraction of the resulting consortium exhibited a high level of xylanase activity, along with carboxymethyl cellulase (CMCase) and filter paper activity (FPA). The main extracellular xylanase was purified and showed stability over a broad pH range, maintaining more than 70% of maximal activity between pH 5.0 and 9.0. Phylogenetic analysis of the consortium using 16S rRNA gene clone library showed that EMSD5 was mainly affiliated with bacteria related to genera *Clostridium*, *Acinetobacter*, *Bacteroides*, *Lysinibacillus* and *Dysgonomonas*. These preliminary findings indicated that EMSD5 was a potential lignocellulolytic consortium, with a high capacity for secreting xylanases. Nevertheless, the inventory of carbohydrate-active proteins used by EMSD5 to deconstruct lignocellulose and the metabolic roles of abundant microbial populations taking part in this process remain to be fully elucidated.

The present study aims to gain a thorough understanding of the plant biomass-decomposing machinery from microbial consortium EMSD5. Therefore, we sequenced and analyzed its metagenome, with a particular focus on lignocellulose degradation-related genes and their taxonomic origins. To complement the metagenome analysis and to characterize the microbial functional diversity, the extracellular metaproteome of EMSD5 cultivated on corn stover was determined by nano liquid chromatography-tandem mass spectrometry (nanoLC-MS/MS) and compared with those of xylan and xylose. In addition, we performed a time course analysis of its extracellular protein profiles during the process of corn stover degradation using a label-free quantitative method.

## Results and discussion

### Secretion of xylanase and endoglucanase by EMSD5 cultivated on corn stover

To determine the suitable time for extracting the metagenomic DNA and extracellular proteins, the timeline of xylanase and endoglucanase secretion by EMSD5 grown on corn stover was analyzed. The xylanase activity in the culture supernatant increased since incubation and reached the highest level of 17.80 U/ml on day 4. The endoglucanase activity was not detected on day 1, but increased afterward to a maximum level of 0.12 U/ml on day 5 (Additional file 1: Figure S1). It seemed that EMSD5 had an enhanced ability to produce extracellular xylanase compared to endoglucanase in the presence of corn stover. From the fifth day, the activities of both xylanase and endoglucanase remained at a fairly steady level, suggesting that enzyme systems of the consortium had reached an optimal state for polysaccharide degradation. The consortium on day 5 was therefore selected for metagenome sequencing and extracellular metaproteome analyses.

### Taxonomic and functional profiles of predicted genes in the metagenome

Metagenome sequencing of EMSD5 generated a total of 864,196 high-quality reads (Additional file 2: Table S1). After de novo assembly, 17,908 contigs longer than 300 bp were obtained. The metagenome of EMSD5 was predicted to contain 48,362 open reading frames (ORFs), with an average length of 714 bp.

**Fig. 1:**
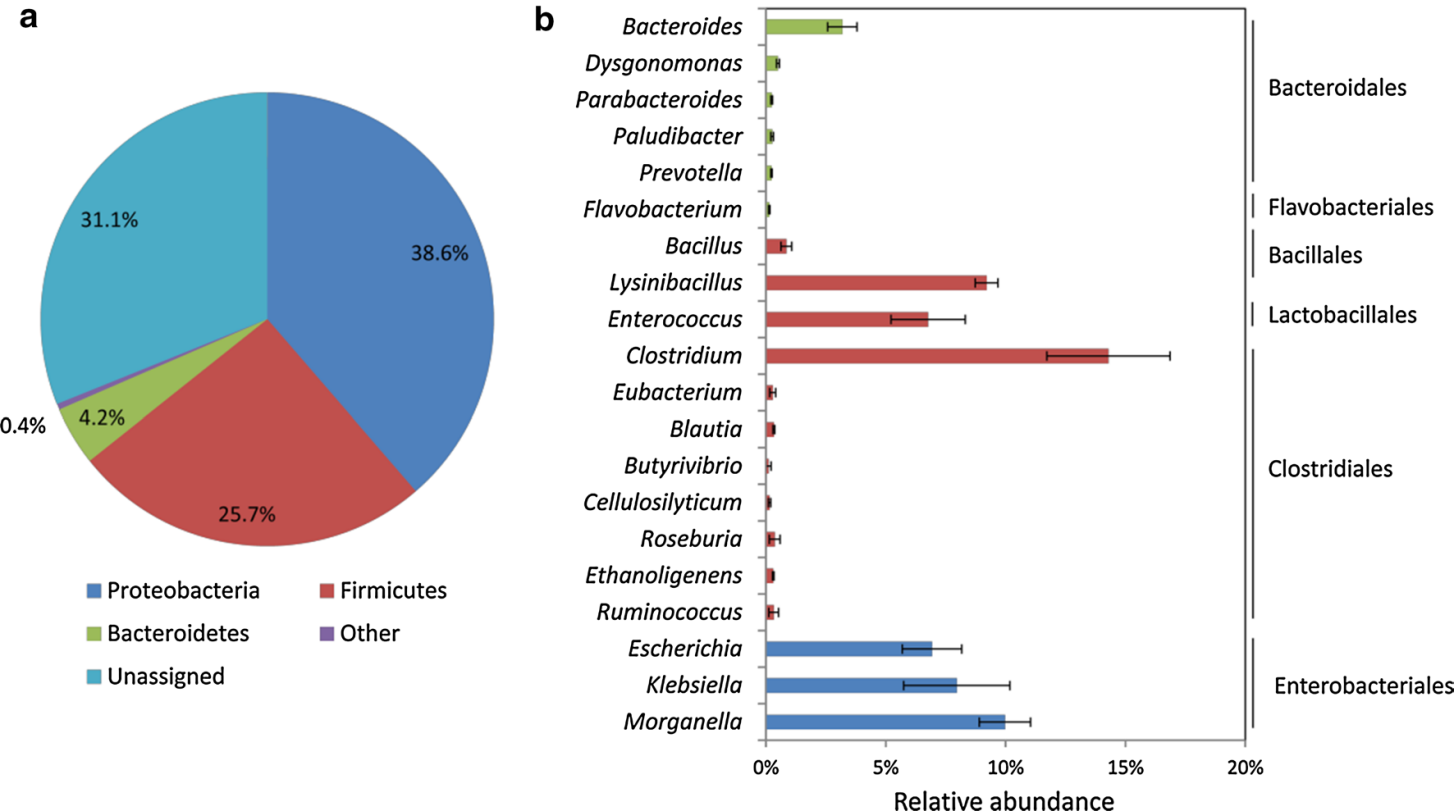
Taxonomic origin of predicted genes at **a** phylum and **b** genus level in the EMSD5 metagenome. Protein-coding genes in the metagenome were taxonomically classified using the MEGAN LCA algorithm.* Error bars* in **b** represent the standard deviation of the mean for three replicate data sets

The taxonomic analysis of all protein-coding genes in the metagenome showed that EMSD5 was predominated by bacteria (99.8% of total sequences), along with very few archaea and eukarya. Around 31.1% of the total sequences could not be assigned to a definite bacterial phylum using the NCBI NT database combined with the MEGAN LCA algorithm, likely representing yet uncharacterized bacteria. Of the assignable protein-coding sequences, 18,689 were affiliated with the phylum *Proteobacteria* (38.6% of total sequences), 12,414 were affiliated with *Firmicutes* (25.7%), and 2017 were affiliated with *Bacteroidetes* (4.2%) (Fig. 1a). Other phyla such as *Spirochaetes*, *Actinobacteria*, *Cyanobacteria*, *Fusobacteria*, *Euryarchaeota*, *Thermotogae* and *Ascomycota* were presented at very low abundances. Compared with other published lignocellulolytic metagenomes, we noticed that the community structure in EMSD5 showed similarity to a microbial community populating biogas reactors [23] and stood in contrast to a rice straw-adapted compost microbial consortium with the phylum *Actinobacteria* being the dominant group [18]. The prevalence of *Proteobacteria* was also found in a sugarcane bagasse microbial community, although it has a much smaller proportion of *Firmicutes* [9]. The metagenomic and 16S rRNA amplicon data sets showed that microbial communities in biogas reactors were frequently affiliated with the phylum *Firmicutes*, which played a significant role in the anaerobic digestion process [12, 23, 24]. Like biogas reactors, the submerged fermentation under static culture conditions in this study provided a relatively anaerobic environment in favor of the growth of anaerobic bacteria such as *Firmicutes*. The exterior of bagasse piles and composts, on the contrary, are considered to be aerobic. At the genus level, 14.3, 10.0, 9.2, 8.0, 7.0, 6.8 and 3.2% of the predicted proteins from the metagenome were assigned to the genus *Clostridium*, *Morganella*, *Lysinibacillus*, *Klebsiella*, *Escherichia*, *Enterococcus* and *Bacteroides*, respectively (Fig. 1b). Species from *Clostridium* were well recognized for their involvement in the decomposition of complex carbohydrates in anaerobic environments [12, 23, 25], and some have previously been shown to possess a variety of genes related to lignocellulose degradation [26, 27]. *Bacteroides* was a typical genus of obligate anaerobes in gut microbial communities [28]. The genera *Morganella*, *Klebsiella* and *Escherichia* of *Proteobacteria* were facultative anaerobes capable of aerobic and anaerobic metabolisms. The community profile of EMSD5 based on metagenomic data set was consistent with previous phylogenetic analysis of the consortium using 16S rRNA gene clone library [22].

The predicted proteins from the metagenomic data set were analyzed for abundant functions using the KEGG and eggNOG databases. The KEGG annotation revealed that 16.9% of total proteins grouped in membrane transport, 12.3% in carbohydrate metabolism and 9.9% in amino acid metabolism (Additional file 3: Figure S2a). Based on the functional categories of COGs, approximately 35.7% of all proteins in the metagenome were poorly characterized, with general or unknown functions (Additional file 3: Figure S2b). Of the proteins with an assigned function, most were associated with amino acid transport and metabolism (11.2%), carbohydrate transport and metabolism (10.3%) and transcription (9.2%). The functional profiles demonstrate that EMSD5 has acquired an enhanced capacity for polysaccharide degradation and sugar uptake in the processing of lignocellulose. It is noteworthy that the functional composition of EMSD5 was similar to that of a switchgrass-adapted microbial consortium and the latter was also enriched from the compost ecosystem [6]. Both microbial communities were abundant in functions associated with carbohydrate and amino acid metabolism. This finding suggests that similarity in metabolic patterns of the above microbial communities could be related to similarity in the original habitats.

### Diversity and taxonomic affiliation of CAZyme genes

Since the plant biomass-degrading capacities of microbial consortia are closely related to genes encoding carbohydrate-active enzymes (CAZymes), the metagenome of EMSD5 was annotated using the dbCAN database. The EMSD5 metagenome harbored a total of 1537 putative CAZyme-encoding genes, 188 of which had two or more domains. The CAZyme repertoire of EMSD5 contained 691 candidate GHs from 75 different families, 348 glycosyltransferases (GTs) from 32 families, 270 carbohydrate esterases (CEs) from 12 families, 56 auxiliary activities (AAs) from 5 families and 41 polysaccharide lyases (PLs) from 11 families in addition to 392 CBMs from 36 families (Additional file 4: Table S2). Analysis of the metagenomic data for CAZymes identified a variety of genes encoding enzymes involved in xylan decomposition. These included six genes encoding GH10 xylanases and five encoding GH11 xylanases for the cleavage of xylan main chains. Xylan component in lignocellulosic biomass has a backbone of xylose residues substituted with various side groups. Complete hydrolysis of xylan substituents requires a suite of debranching enzymes. In EMSD5, β-xylosidases, α-l-arabinofuranosidases and α-glucuronidases were predicted in high abundances. The metagenome of EMSD5 contained 33 genes encoding GH43 enzymes with β-xylosidase or α-l-arabinofuranosidase activities. Also identified were three genes encoding α-glucuronidases, of which two were in the GH115 family and one in the GH67 family. The xylan-degrading capacity of EMSD5 was further complemented by the presence of a number of genes from families CE1, CE2, CE3, CE4 and CE7. Enzymes from these families were mostly assigned to acetyl xylan esterases based on sequence similarity, which have been demonstrated to facilitate xylan solubilization by removing acetyl substituents [29].

The complete hydrolysis of cellulose involves the synergistic action of three different classes of cellulases: endo-1,4-β-glucanases, exo-1,4-β-glucanases and β-glucosidases. The metagenome of EMSD5 harbored a full suite of genes for cellulose degradation, including 28 genes encoding GH5 and GH9 endoglucanases, 22 genes encoding putative GH3 β-glucosidases and 1 gene encoding GH48 cellobiohydrolase. With respect to lignin degradation, four genes encoding class II peroxidases from AA2 family were identified in the metagenome. When examining the family distribution of genes encoding GHs, we noticed that the most abundant GH family was GH13 with 62 predicted genes. The presence of a high number of GH13 genes indicated that EMSD5 had great potential to utilize α-glucan like starch and was a promising source of amylolytic enzymes that can be used in the food and starch-based biofuel industries.

The metagenome of EMSD5 was particularly rich in genes encoding glycoside hydrolases with multiple CBM domains. Strikingly, of the 126 CBM-containing enzymes, 65 were associated with a single CBM (51.6%) and 61 were equipped with multiple CBMs (48.4%). Polysaccharide-hydrolyzing enzymes bearing multiple CBMs are found most frequently in thermophilic bacteria [30]. The recognized role of CBMs is to increase enzyme concentrations on the surface of substrates by targeting and binding to substrates, but the biological significance of multiple CBMs is obscure. It has been suggested that multiple CBMs may counteract the loss of binding affinity between thermophilic enzymes and their substrates at elevated temperatures [30]. During a typical composting process, compost microbiota experience a thermophilic phase (above 50 °C) for about one week and much longer mesophilic phases (20–50 °C) [6, 31]. It remains to be seen whPaenibacillusether compost-derived microbial consortia employ a similar strategy to enable strong binding to insoluble polysaccharide substrates.

In some anaerobic bacteria, the strong interactions between the cells and insoluble polysaccharide substrates are mediated by cellulosomes, and the cohesin-dockerin interaction is crucial for the assembly of cellulosome complexes [32]. Currently, 11 species in the phyla *Firmicutes* and *Bacteroidetes* have been reported to be cellulosome-producing bacteria [33, 34]. Although representatives of *Firmicutes* and *Bacteroidetes* were dominant in EMSD5, no genes encoding putative cellulosome-associated modules, such as cohesins and dockerins, were identified in the metagenome of EMSD5. The absence of cellulosomes in cellulolytic systems is also observed in the metagenomic studies of *Firmicutes*- and *Bacteroidetes*-dominating wallaby foregut [35] and yak rumen microbiome [36]. Analysis of the taxonomic profile of candidate CAZymes in EMSD5 at the species level revealed that the majority of these enzymes originated from *Firmicutes* and *Bacteroidetes* bacteria which produced free enzyme systems for the degradation of cellulose and hemicellulose in biomass. It has been reported that free enzymes are more effective at degrading pretreated lignocellulosic biomass than cellulosomes, while cellulosomes have faster digestion rates on purified cellulose than free cellulases [37]. Since the microbial consortium EMSD5 was adapted to corn stover rather than cellulosic substrates, it seems reasonable that the plant biomass-decomposing enzyme systems of EMSD5 mainly comprised free complementary enzymes. This may provide EMSD5 with more flexibility to regulate the expression of enzymes and to produce enzyme combinations with different substrate preference.

To relate metabolic functions to abundant community members, all genes associated with plant biomass breakdown were taxonomically classified. CAZyme-encoding genes were mostly distributed within the phyla *Firmicutes*, *Proteobacteria* and *Bacteroidetes*, but the number of CAZyme genes varied across bacterial genera (Additional file 5: Figure S3a). Around 45.1% of these genes were encoded by the genomes of representatives of *Firmicutes*, followed by *Proteobacteria* (24.9%) and *Bacteroidetes* (21.0%). In addition to *Clostridium* (22.0%) of the phylum *Firmicutes*, the CAZyme genes were also mainly present in *Bacteroides* (12.2%) of the phylum *Bacteroidetes* as well as *Escherichia* (11.0%) and *Klebsiella* (10.1%) of the phylum *Proteobacteria*. Members of *Firmicutes* were predominant in the number of potential GHs, CEs, AAs and associated CBMs, whereas *Proteobacteria* had more GTs and *Bacteroidetes* abounded with PLs (Additional file 5: Figure S3b). The predominance of these lignocellulolytic community members in EMSD5 was closely related to its capacity of degrading lignocellulosic biomass. These results also support the feasibility of using enrichment cultures to obtain microbial consortia with desired properties from compost habitats.

**Fig. 2:**
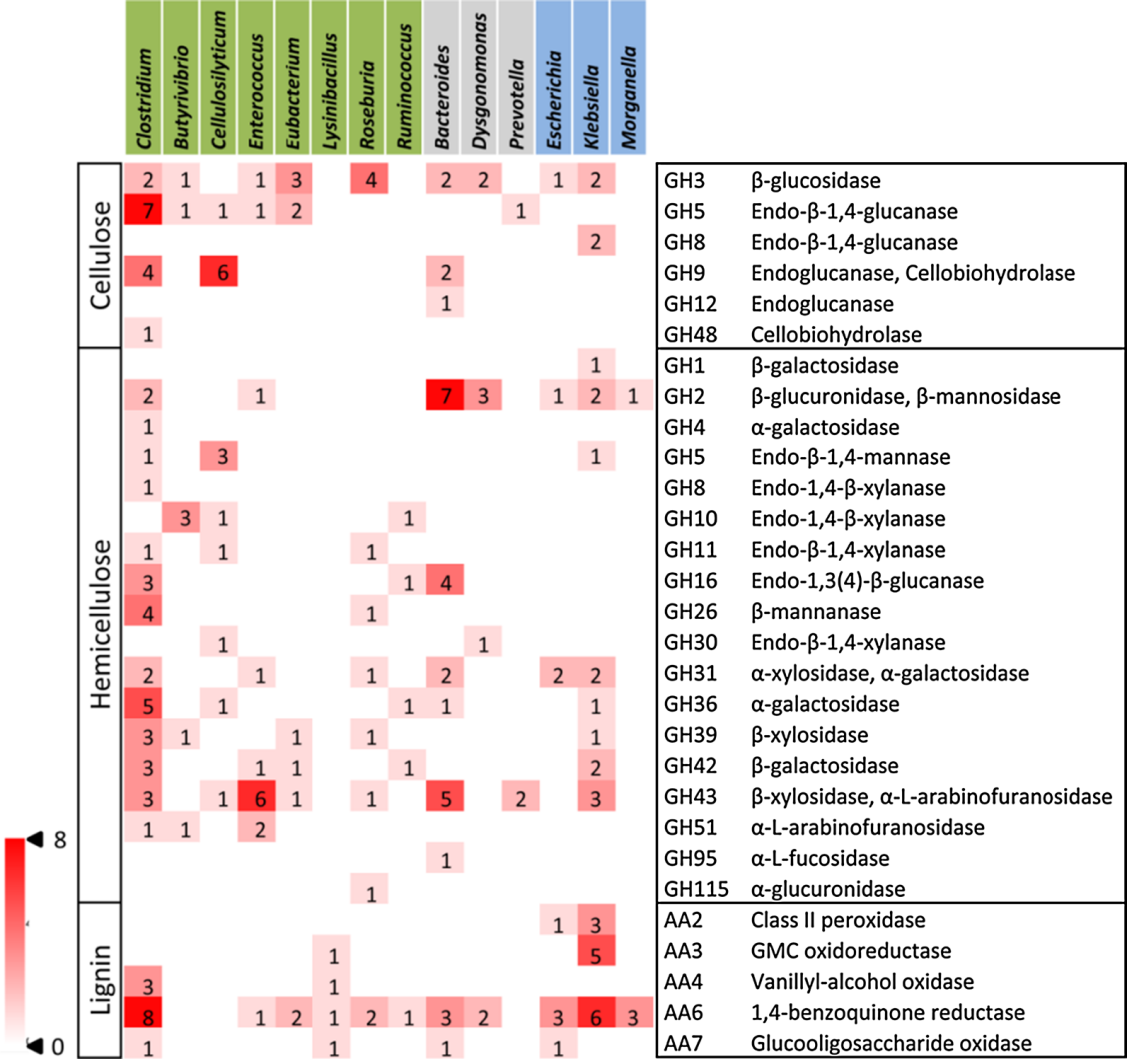
Heat map showing the distribution of glycoside hydrolase (GH) and auxiliary activities (AA) families in the abundant genera. CAZy families are grouped according to their activities on major components of plant cell walls. Only GH families targeting (hemi)cellulose and AA families targeting lignin are taken into account. Only genera with ten or more GHs are presented. *Firmicutes* are depicted in *green*, *Bacteroidetes* in *gray* and *Proteobacteria* in *blue*

A detailed investigation of the taxonomic distribution of GHs showed that 8 of 14 genera with the most GHs in EMSD5 were from *Firmicutes*, compared to 3 and 3 from *Bacteroidetes* and *Proteobacteria*, respectively (Fig. 2). Our results showed that three bacterial phyla encoded varying numbers of endo-acting and oligosaccharide-degrading cellulases, and *Firmicutes* bacteria were predicted to produce the GH9 and GH48 enzymes (cellobiohydrolases). In fact, most of the putative enzymes belonging to cellulolytic GH families 3, 5, 8, 9, 12 and 48 originated from members of *Firmicutes*, especially *Clostridium* and *Cellulosilyticum*. Notably, the number of genes coding for cellulolytic GH enzymes affiliated with *Firmicutes* was threefold and sixfold higher than that with *Bacteroidetes* and *Proteobacteria*, respectively. A higher number of hemicellulolytic GH genes with wider distribution were also observed in members of *Firmicutes*. With regard to lignin degradation, both *Firmicutes* and *Proteobacteria* possessed 22 genes encoding AA family enzymes, but only 4 AA2 genes were assigned to *Escherichia* and *Klebsiella* of the phylum *Proteobacteria*. These results indicated that members of *Firmicutes* harbored an enriched catalog of genes encoding potential cellulases, hemicellulases as well as peroxide-generating enzymes, while *Proteobacteria* was richer in gene families associated with direct ligninolysis. These observations suggest that microbial populations may have different metabolic niches within the consortium in the decomposition of lignocellulosic biomass. Specifically, members of *Firmicutes* and *Bacteroidetes* were assumed to act mainly on the complex polysaccharide such as cellulose and hemicellulose, whereas *Proteobacteria* was more tuned to degrading lignin.

### Extracellular metaproteomes induced by different substrates

The metagenome analysis showed that EMSD5 had a diversity of genes and community members involved in plant biomass deconstruction. To investigate the actual expression of lignocellulolytic enzymes, nanoLC-MS/MS analysis was used to identify the secreted proteins by EMSD5 cultivated in minimal media containing corn stover as the sole substrate. Considering that the metagenome of EMSD5 encoded a diverse set of xylan deconstruction-related enzymes, xylan and its constituent monomeric sugar xylose were chosen as comparisons to corn stover as they had different structural complexity.

**Fig. 3:**
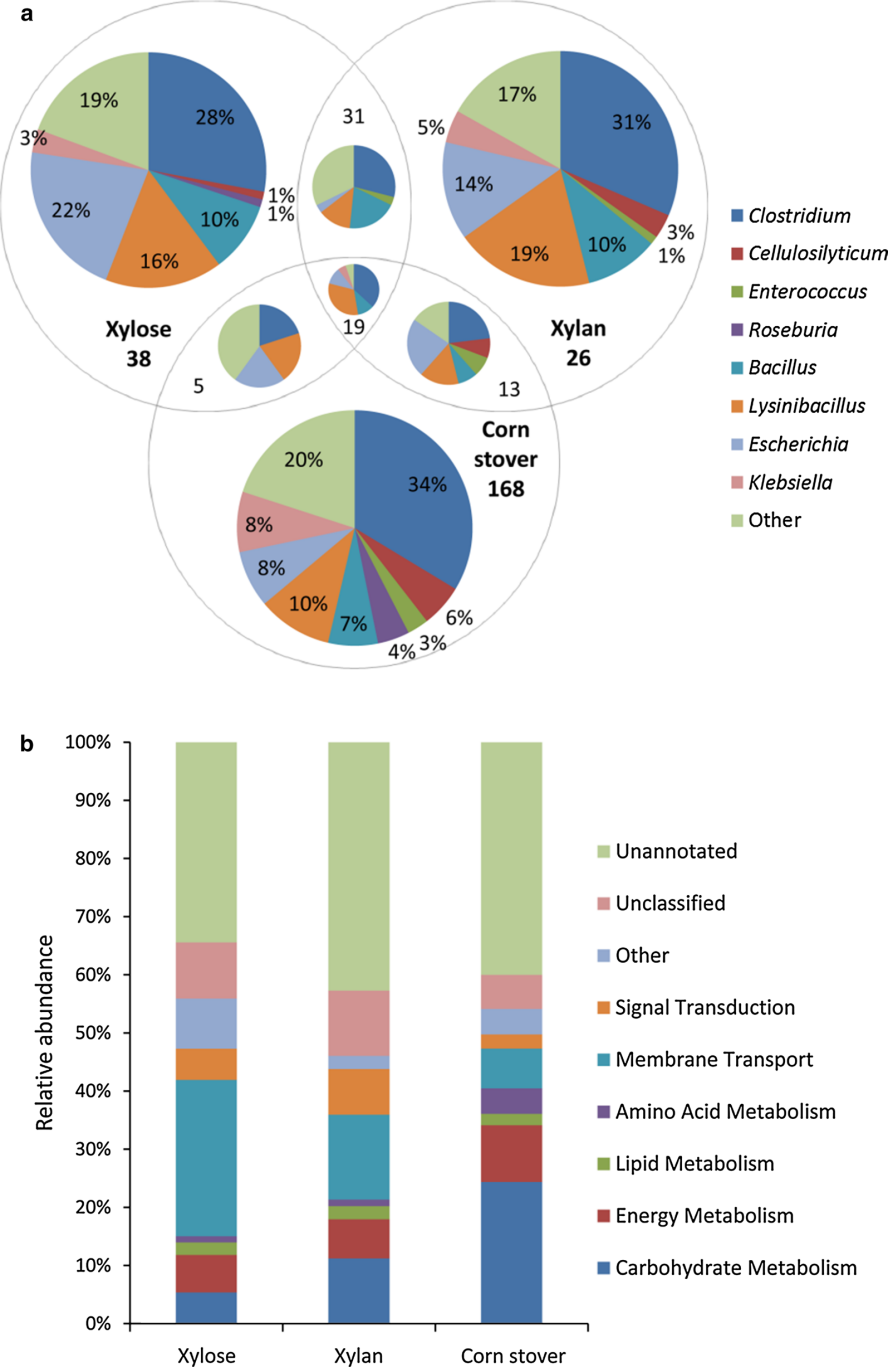
Extracellular proteins produced by EMSD5 during growth on corn stover, xylan and xylose. **a** Taxonomic assignment and **b** functional annotation of proteins detected in culture supernatants of three carbon sources. Functional predictions of identified proteins are based on the KEGG orthology system

In total, 205 proteins were identified on corn stover, 89 proteins on xylan and 93 on xylose (Additional file 6: Table S3). Of these proteins, 168 were exclusively found on corn stover, while 38 and 26 were unique to xylose and xylan, respectively. Besides, 24 proteins were common between corn stover and xylose, 32 between corn stover and xylan, and 50 between the xylan and xylose. 19 proteins were shared by all three carbon sources (Fig. 3a). For all three substrates, the extracellular proteins mainly originated from the phyla *Firmicutes* and *Proteobacteria*. Interestingly, the percentage of corn stover-specific proteins affiliated with *Firmicutes* (71.7%) was much higher than that affiliated with *Proteobacteria* (20.5%), while predicted proteins from *Proteobacteria* were more abundant in the metagenome. This discrepancy may have arisen because only the extracellular proteins of the consortium, which were closely related to biomass deconstruction, were collected for metaproteome analysis. At the genus level, however, the taxonomic profiles varied with carbon sources. The percentages of detected proteins from *Clostridium* (34%), *Cellulosilyticum* (6%), *Roseburia* (4%) and *Enterococcus* (3%) were higher on corn stover than xylan and xylose, while those affiliated with *Bacillus*, *Lysinibacillus* and *Escherichia* showed higher abundance on xylose. Additionally, 8% of the proteins detected on corn stover were affiliated with *Klebsiella* in comparison to 3–5% on xylose and xylan, respectively. *Clostridium*, *Cellulosilyticum* and *Roseburia* are considered as potential biomass-decomposing bacteria. The genus *Clostridium* belongs to the core set of microbiome in biogas plants responsible for the degradation of complex carbohydrates [12]. Several strains from the species *Clostridium clariflavum* have been isolated from thermophilic compost [38], and physiological characterization reveals that they are highly efficient in the breakdown of cellulose and/or hemicellulose [39, 40]. *Cellulosilyticum ruminicola* H1, an anaerobic bacterium, has been reported to be an active degrader of plant fibers in yak rumen [41]. This strain is capable of growing vigorously on natural plant biomass as well as on a variety of (hemi)cellulosic polysaccharides, including cellulose, xylan and mannan. The major xylanolytic species in the human gut microbiota are assigned to *Roseburia* and *Bacteroides* [42], and their isolates display high xylanase activity on oat spelt xylan [43]. Although it has not previously been associated with plant biomass degradation, species from *Enterococcus* have been shown to be able to metabolize secondary compounds in plants, such as alkaloids and latex [44]. Thus, our results revealed a shift in the community structure of EMSD5, i.e., the presence of corn stover fostered the prevalence of bacteria involved in plant polysaccharide breakdown.

The metaproteomic results showed that the functional composition of secreted proteins from EMSD5 differed substantially between the three carbon sources. About 45.9% of all proteins detected in the corn stover-specific metaproteome had no KEGG annotation (i.e., unannotated and unclassified), representing proteins with unknown functions (Fig. 3b). Most of the assignable proteins were involved in carbohydrate metabolism (24.4%), energy metabolism (9.8%) and membrane transport (6.8%). On xylan, 46.1% of the proteins have a putative function predicted by KEGG. The most abundant function was membrane transport (14.6%), followed by carbohydrate metabolism (11.2%). On xylose, 26.9–5.4% of the proteins were related to membrane transport and carbohydrate metabolism, respectively, whereas 44.8% showed no predicted functions. Evidently, exposure to corn stover resulted in increased expression of proteins involved in carbohydrate processing.

### Carbohydrate-active enzymes in three extracellular metaproteomes

**Fig. 4:**
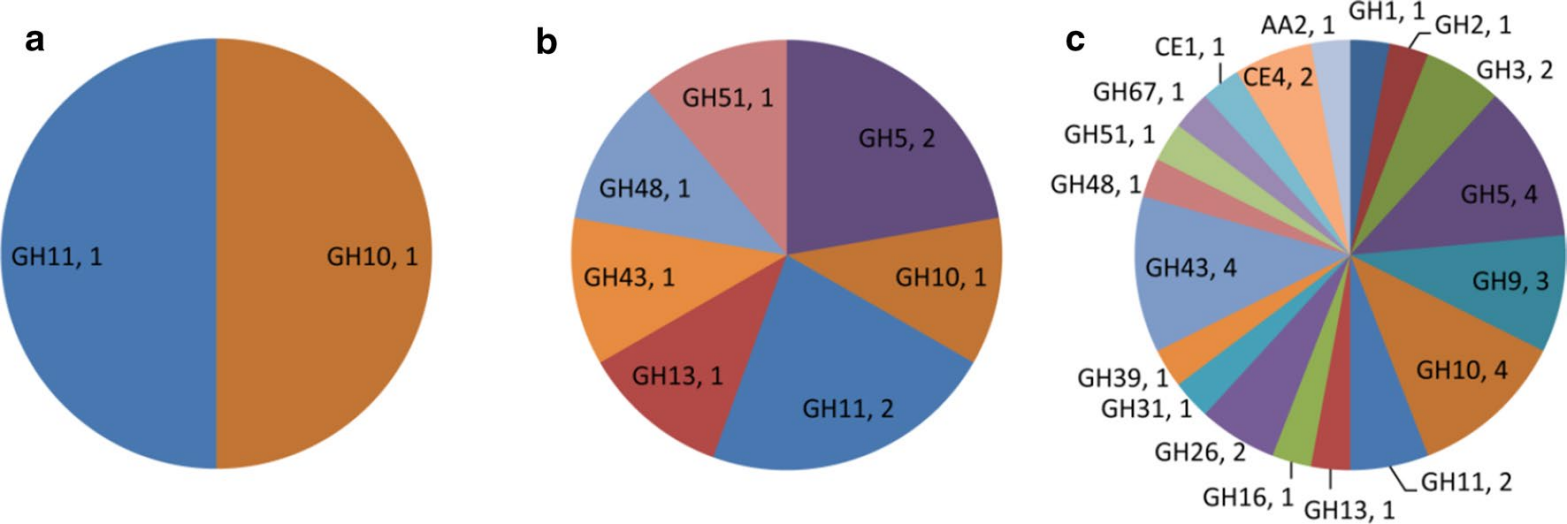
Distribution of CAZy family proteins identified in culture supernatants of **a** xylose, **b** xylan and **c** corn stover. Family annotations of the proteins are based on the carbohydrate-active enzyme database (CAZy)

To distinguish the degradative enzyme systems on different substrates, the metaproteomic data sets were analyzed with respect to lignocellulolytic proteins and with respect to their taxonomic origins. 34 of the 205 proteins (16.6%) detected on corn stover were involved in the deconstruction of plant biomass, while there were only 9 (10.1%) and 2 (2.2%) in xylan- and xylose-containing media, respectively (Fig. 4). On corn stover, proteins of the CAZy families active against xylan, such as GH10, GH11, GH39, GH43, GH51, GH67, CE1 and CE4, were the most abundant group, accounting for half of the total CAZymes. The cellulolytic enzyme systems of EMSD5 on corn stover consisted of five endoglucanases (GH5 and GH9), two cellobiohydrolases (GH9 and GH48) and one β-glucosidase (GH3). Also identified on corn stover were GHs targeting other polysaccharides, such as GH26 (β-mannanase), GH31 (α-xylosidase), GH2 (β-glucuronidase), GH13 (α-amylase) and GH16 (endo-1,3(4)-β-glucanase). In contrast to the broad spectrum of hydrolytic enzymes, only one AA2 protein was found to be potentially involved in lignin degradation. This may be due to our current lack of understanding of bacterial enzymes associated with lignin deconstruction. Most of these proteins were exclusively detected on corn stover. The extensive complement of CAZymes expressed in the presence of corn stover reflects the extraordinary ability of EMSD5 to degrade a diversity of polysaccharides available in this complex substrate.

A unique feature of the corn stover-induced metaproteome was the enrichment of multi-modular CAZymes (Additional file 7: Table S4). All of the CBM-containing enzymes found on corn stover were predicted to be extracellularly secreted based on the presence of signal peptides, with the exception of a GH11 xylanase (ID: 46506) and a CE4 acetyl xylan esterase (ID: 46503). The majority of these enzymes had modular architecture containing a single catalytic domain and multiple CBM domains. The cellobiohydrolase (ID: 45317) from *Clostridium saccharoperbutylacetonicum* contained triplicate N-terminal CBM4, a CBM30, a GH9 and a C-terminal CBM3. Although it shared 50% amino acid sequence identity with a putative xylanase from *Eubacterium cellulosolvens* (NCBI accession: WP004603161.1), the enzyme (ID: 48211) had a CE1 domain in addition to an N-terminal GH10 domain and two CBMs. It is possible that this enzyme may have a bifunctional role in the deconstruction of xylan. The α-amylase (ID: 19270) from *Clostridium saccharoperbutylacetonicum* had an N-terminal GH13 in tandem with six CBM26 domains. The β-mannanase (ID: 46460) from *Cellulosilyticum lentocellum* and xylanase (ID: 44363) from *Roseburia intestinalis* harbored seven and six domains, respectively. Such multi-modular organization confers a strong interaction between free enzymes and the substrates, which is critical for efficient hydrolysis of insoluble polysaccharides [45]. In contrast to these multi-modular enzymes observed in EMSD5, the cellulolytic enzyme systems of the fungus *T. reesei* and the bacterium *T. fusca* mainly comprise six or seven hydrolases containing a single catalytic domain with one CBM domain [1, 2]. It has been demonstrated that multi-modular enzymes have a mode of cellulose digestion distinct from that of fungal enzymes with a single CBM. Typical fungal enzymes from *T. reesei* ablate the surface of cellulose microfibril bundles. In contrast, multi-modular enzymes fully degrade cellulose microfibrils at binding regions and generate cavities in the substrate layers due to significantly lower off-rates from substrates [5]. Defining the functions of these tandem CBMs in intact enzymes can provide a better insight into the mechanism of polysaccharide deconstruction by EMSD5.

Growth on xylan induced the expression of three xylanases, one β-xylosidase and one α-l-arabinofuranosidase. Unexpectedly, two of the GH5 endoglucanases, one GH48 cellobiohydrolase and one GH13 α-amylase detected on corn stover were also present in xylan-containing medium. It seemed that exposure to xylan led to the expression of hemicellulases, as well as these cellulose- and starch-degrading enzymes even in the absence of their substrates. It should be noted that, of the nine GH proteins identified on xylan, eight were also expressed on corn stover, except for a GH10 xylanase (ID: 41289). Expression of the extracellular (hemi)cellulolytic enzymes by community members was repressed by xylose. The only two CAZymes identified in xylose culture were classified as GH10 and GH11 xylanases, possibly due to a low level of constitutive expression. The above observations indicate that the expression of most degradative enzymes in EMSD5 was substrate dependent. Complex lignocellulosic substrates, such as corn stover, contain a wide range of polysaccharides that induce the expression and secretion of a high diversity of CAZymes. In contrast, the extracellular degradation of simple polysaccharide xylan requires much fewer glycoside hydrolases, and xylose completely represses their expression. The differential response to the composition of biomass substrates has also been observed in recent metaproteomic analysis of a wheat straw-adapted consortium [19], but some substantial differences between the two consortia still stood out. The majority of secreted GHs in the presence of wheat straw were involved in the degradation of hemicellulose (GH10, GH43, GH51 and GH95) and α-glucan polysaccharides (GH13), while cellulolytic enzymes mainly comprised GH3 β-glucosidases. Despite the repression of hemicellulases and pullulanases secretion by xylose, oligosaccharide-degrading enzymes (GH1 and GH3) and GTs were detected. In contrast to our results, no enzymes involved in polysaccharide deconstruction were found on xylan. Most of the carbohydrate-active enzymes in the wheat straw metasecretome were taxonomically affiliated with *Sphingobacterium* and *Klebsiella* species. These differences between the two consortia in the induction of plant biomass-degrading enzymes might mirror the difference in their taxonomic compositions, as well as in the lignocellulolytic capacities of the community members.

The metaproteome analysis in this study focused on the extracellular proteins in the supernatants, as proteins associated with lignocellulose deconstruction were often observed in this fraction. It should be noted that biomass-degrading enzymes, especially enzymes with multiple CBMs, may adsorb to the insoluble lignocellulosic substrates. Previous work has shown that considerable amounts of cellulolytic enzymes secreted by a microbial community EMSD13 were bound to the substrate sugarcane bagasse [46]. The cellobiohydrolase (CBH) activity in the substrate eluents was higher than that in the supernatant fraction, suggesting a strong binding of CBHs to the insoluble substrates. Zymogram analysis showed distinct protein band patterns for the substrate eluents and supernatants. At least six protein bands were present exclusively in the substrate eluents. Identification of the substrate-bound proteins will provide a more comprehensive view of lignocellulose deconstruction by EMSD5.

To affirm the metaproteome results, the (hemi)cellulolytic enzyme activities induced by three carbon sources were measured and compared. Consistent with the metaproteome analysis, growth on corn stover produced significantly higher levels of all tested cellulolytic and xylanolytic activities in comparison to xylan and xylose (Additional file 8: Figure S4). In particular, the consortium exhibited an extraordinary capacity for producing xylan-degrading enzymes, especially xylanases when corn stover was used as the substrate. The specific activities of β-glucosidase and xylan esterase were detected only in the presence of corn stover.

Based on the taxonomic annotation, the majority of cellulolytic enzymes, including four endoglucanases and two cellobiohydrolases, were secreted by *Clostridium* and *Cellulosilyticum*, establishing these two genera as the main players responsible for cellulose depolymerization (Additional file 7: Table S4). Besides, a GH5 endoglucanase (ID: 46607) was assigned to *Eubacterium cellulosolvens*, and the only β-glucosidase detected was tracked to *Bacteroides coprosuis*. A much more diverse suite of consortium members and their enzymes were involved in hemicellulose deconstruction. Xylanases of GH10 and GH11 were found to be affiliated with *Cellulosilyticum*, *Roseburia*, *Ruminococcus*, *Lachnoclostridium* and *Butyrivibrio*. The four β-xylosidases were present in *Flavobacterium*, *Clostridium*, *Cellulosilyticum* and *Sphaerochaeta*. Microbial and functional diversity was also observed in accessory enzymes involved in the removal of xylan side chains, such as arabinose, glucuronic acid and acetyl groups. In EMSD5, *Enterococcus* and *Klebsiella* acted as the source of α-l-arabinofuranosidases, xylan esterase activities were mainly derived from *Clostridium*, *Pseudobutyrivibrio* and *Enterococcus*, and GH67 α-glucuronidase was secreted by *Paenibacillus*. It is generally accepted that the complete breakdown of xylan requires the action of both xylanases and debranching enzymes such as α-l-arabinofuranosidases, α-glucuronidases and acetyl xylan esterases [47]. In this context, the above genera constituted an indispensable subgroup of microbial populations which contributed to the complete degradation of xylan. In addition, three putative β-mannanases were affiliated with *Roseburia intestinalis*, *Cellulosilyticum lentocellum* and *Clostridium clariflavum*, respectively. The only AA2 protein was, according to PSI-BLAST and KEGG annotation, a catalase/peroxidase from *Escherichia coli* (100% coverage and 100% identity). Thus, a wide assortment of enzymes with complementary activities was produced by physiologically diverse members and acted in a synergistic way, suggesting a cooperative relationship between community members in the degradation of lignocellulosic biomass. These results also supported the fact that the phylum *Firmicutes* was the predominant group responsible for the deconstruction of plant polysaccharide in EMSD5, as observed in the metagenome data. Most of the degradative enzymes acting on cellulose, xylan and mannan were secreted by members from this phylum, such as *Clostridium* and *Cellulosilyticum*, among others. Previous studies have revealed that members from these genera were capable of secreting a comprehensive set of cellulolytic and hemicellulolytic enzymes [48–50], or were highly efficient in the breakdown of biomass polysaccharides [40]. Our results not only provided evidence for the lignocellulolytic capacities of these genera, but also identified their specific functions and synergistic relations within the community in plant biomass deconstruction.

### Dynamics of corn stover-induced metaproteome over time

**Fig. 5:**
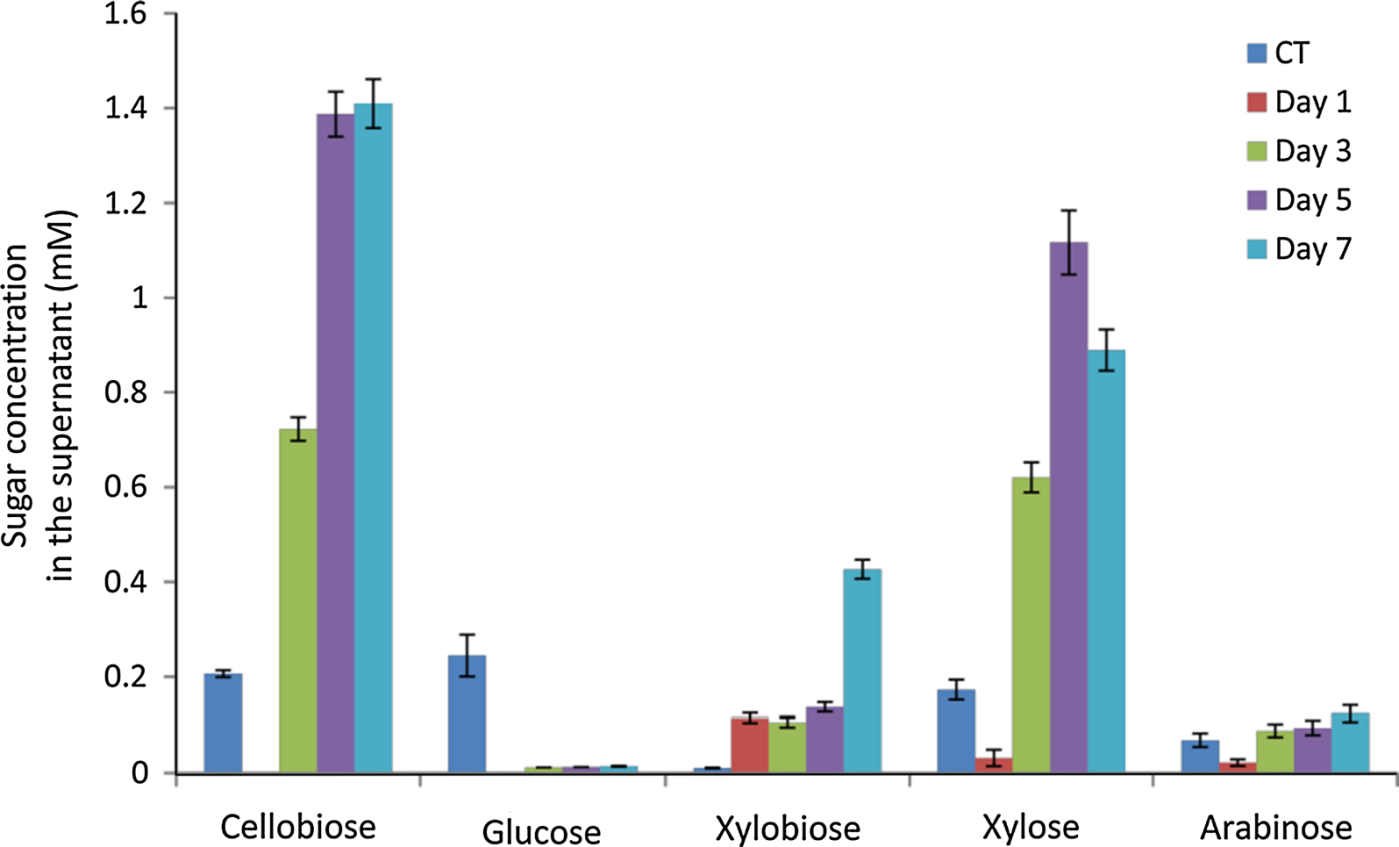
Free sugars in culture supernatants of corn stover-containing medium. The sugar content of culture supernatants is analyzed by HPLC after 1, 3, 5 and 7 days of cultivation. The control sample before inoculation is designated as CT. The data represent the mean of three replicates and the *error bars* indicate standard deviations from the mean values

The above results showed that EMSD5 employed a wide spectrum of lignocellulolytic enzymes to break down complex lignocellulosic biomass. To understand the differential roles of these enzymes during corn stover degradation, we conducted a label-free quantitative proteomics analysis of time course metaproteomes. The sugar content of the culture supernatants over time was analyzed by HPLC to determine the time points for the quantitative analysis. Before inoculation, multiple disaccharide and monosaccharide sugars, including cellobiose, glucose, xylose and arabinose were present in the supernatant fraction of corn stover-containing minimal media (Fig. 5). After 1 day of cultivation, all free sugars in the supernatant were depleted. However, the release of xylobiose indicated that degradation of easily accessible xylan fraction had begun to take place. By day 3, significant increase was observed in the concentrations of xylose, arabinose and cellobiose, while xylobiose level remained relatively unchanged. This observation demonstrated that all the major polysaccharide components of corn stover had been enzymatically broken down by this time point. Interestingly, glucose concentration remained below detectable level during the course of incubation. This observation could be due to a faster rate of uptake than degradation by consortium members. At the end of cultivation (day 7), concentrations of all free sugars, except xylobiose, did not increase further indicating that polysaccharide deconstruction and sugar uptake had reached a balance. Based on these observations, extracellular protein fractions were sampled after 1, 3 and 7 days of cultivation, respectively.

**Fig. 6:**
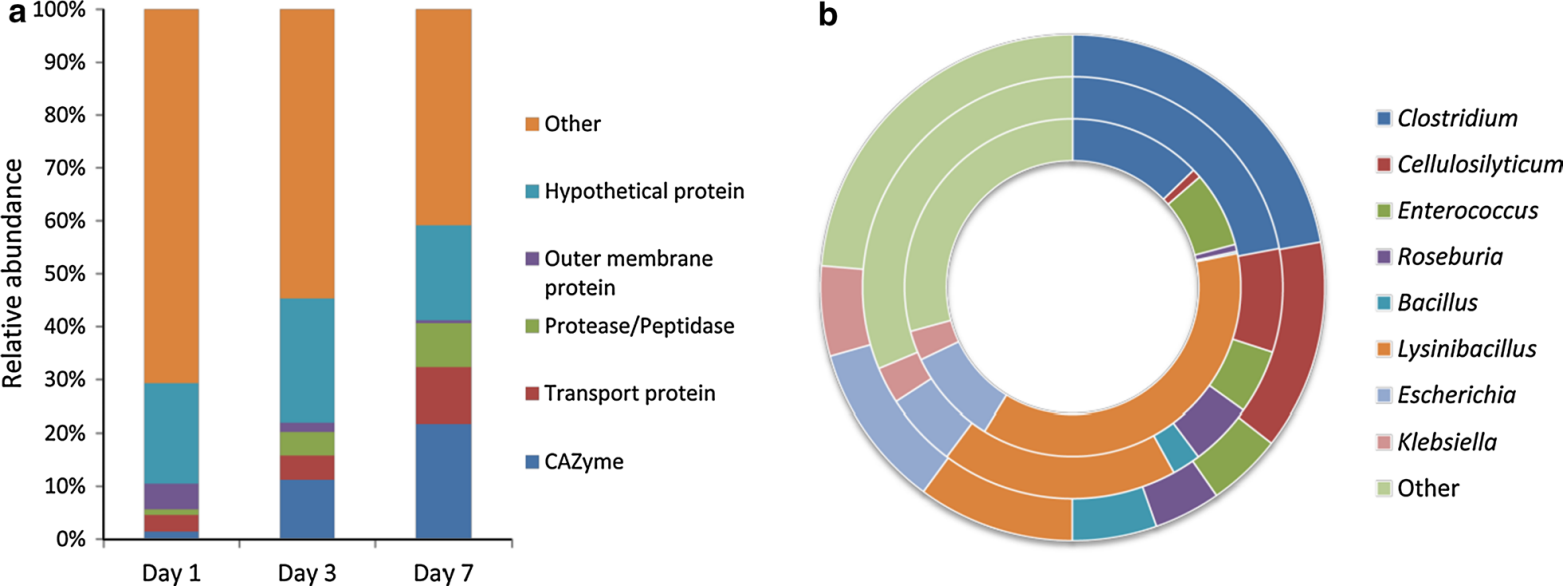
Corn stover-induced extracellular proteins on days 1, 3 and 7. **a** Functional classification and **b** taxonomic distribution of proteins identified in culture supernatants on days 1, 3 and 7, respectively. Percentages of proteins from each category are calculated from normalized LFQ intensities. The *inner circle* represents taxonomic distribution of proteins detected on day 1, followed by day 3 in the *middle* and day 7 in the *outer circle*

The extracellular protein complements at each time point were determined using LC-MS/MS, followed by label-free quantification by normalized LFQ intensity, a method based on MS peak area intensity of detected peptides. A full list of the expression levels of 255 identified proteins is included in Additional file 9: Table S5. A total of 129, 226 and 232 proteins were identified in the culture supernatants of corn stover-containing media on days 1, 3 and 7, respectively. Functional annotation of the secreted proteins revealed that the percentage of CAZymes (based on LFQ intensity) increased with the cultivation time (Fig. 6a). On day 1, CAZymes represented approximately 1.5% of total proteins detected. By day 3, expression of CAZymes encompassed 11.2% of total proteins, indicating a strong overrepresentation of the CAZymes, as they represented only 3.2% of the predicted proteins in the metagenome. At the end of cultivation, the proportion of CAZymes was further increased to 21.8% of total detected proteins. The percentage of transport proteins, potentially associated with importing various oligosaccharides and simple sugars, rose from 3.1 to 10.6% over the incubation period. An increased expression pattern was also observed in protease and peptidase (from 1.0 to 8.3%). The proportion of hypothetical proteins rose to 23.3% on day 3, followed by a decline thereafter. In contrast, the percentage of other proteins decreased from 70.7 to 40.8%. The increased abundance of functional proteins related to carbohydrate processing and transporting revealed that carbohydrate metabolism pathways in EMSD5 were activated to deconstruct corn stover. The extracellular proteins were also taxonomically classified. The relative abundance of proteins affiliated with *Clostridium* increased from 12.8% on day 1, to 22.1% on day 3 and then remained relatively steady (Fig. 6b). The percentage of proteins from *Cellulosilyticum* increased from 0.9 to 13.4% over the course of 7 days, which could be attributed in part to the increased secretion of xylanases and endoglucanases by *Cellulosilyticum*. This shifting pattern of community structure showed that members in relation to plant polysaccharide degradation became dominant as cultivation time advanced, which was responsive to the presence of complex polysaccharides.

**Fig. 7:**
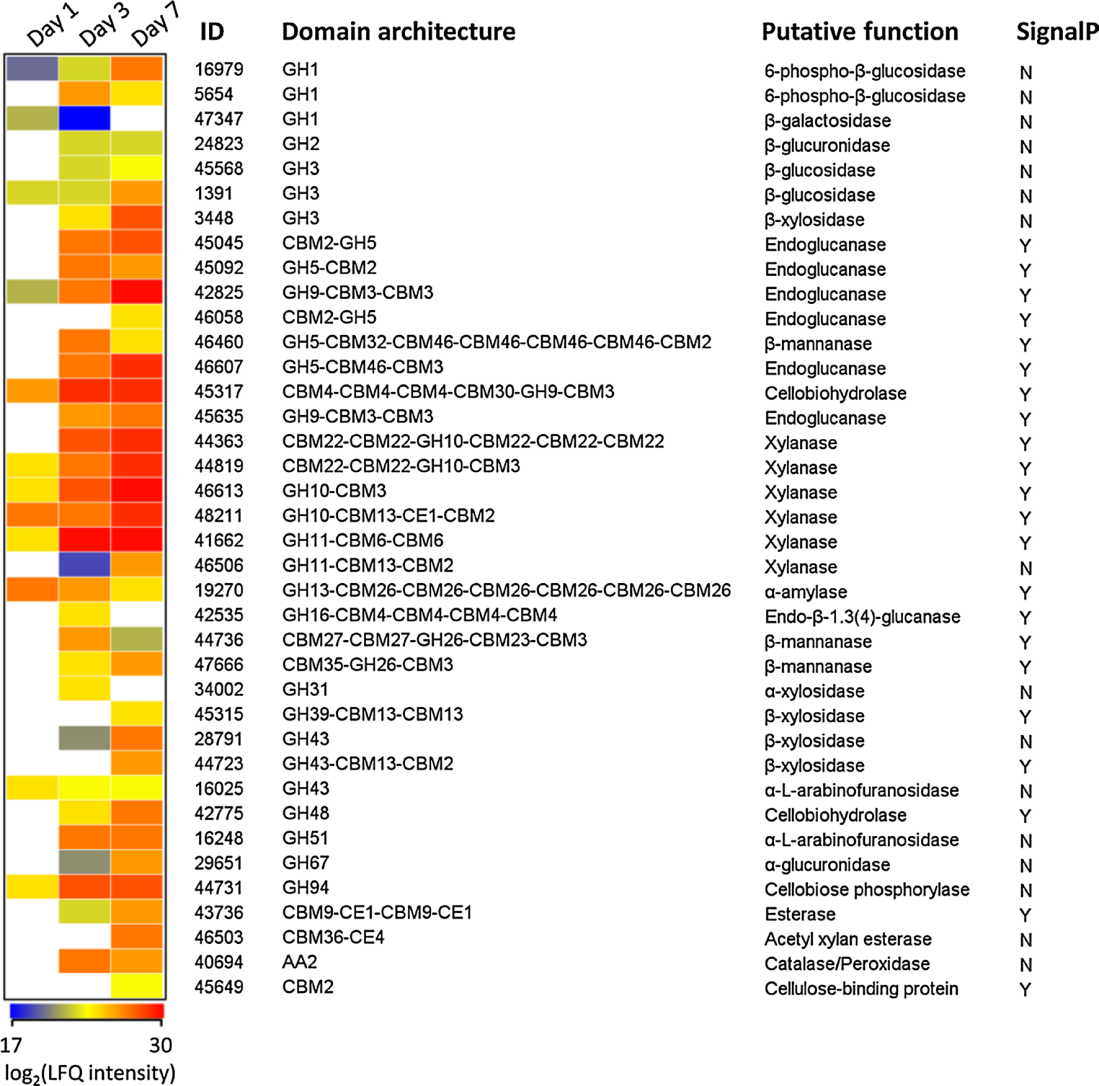
Time course protein abundance of CAZymes produced by EMSD5 during growth on corn stover. Family information is obtained from dbCAN annotation. Putative functions are analyzed using PSI-BLASTP and KEGG annotation. Prediction of signal peptides is based on SignalP analysis. The *scale below the map* indicates log_2_-transformed LFQ intensities. Undetected CAZymes are shown in *white*

The temporal expression patterns of plant biomass-decomposing enzymes were investigated more closely. There were only 12 CAZymes detected on the first day of cultivation (Fig. 7). The secretion of four different xylanases was in line with the detection of xylobiose on day 1, supporting a model in which the xylan fraction of corn stover was preferentially degraded by EMSD5. A GH13 α-amylase (ID: 19270), a GH10 xylanase (ID: 48211) and a GH9 cellobiohydrolase (ID: 45317) were present in the highest abundances, whereas the only endoglucanase (ID: 42825) was expressed at comparatively low levels at the beginning of cultivation. The cause for the high abundance of α-amylase was unclear. A recent transcriptomic study suggested that genes encoding amylases in *Paenibacillus *could be induced by xylan [51]. This was, however, unlikely in our case, because the protein abundance of the α-amylase decreased significantly over the cultivation period, as detailed below. It seemed likely that the high expression level of the α-amylase was triggered by the presence of starch, a non-structural polysaccharide in corn stover. The amorphous starch would be readily accessible to enzymes compared to xylan and cellulose. It appeared that a small subset of degradative enzymes played a major role in the initial polysaccharide deconstruction.

By day 3, a much more diverse set of CAZymes were represented in the supernatant, including proteins annotated as GH3 and GH43 β-xylosidase, GH26 β-mannanase, GH48 cellobiohydrolase, GH51 α-l-arabinofuranosidase and CE1 esterase. All the GHs identified on the first day showed increased abundance on day 3, except the GH13 α-amylase displaying a reverse trend. The most abundant enzyme was a xylanase (ID: 41662) with a GH11 domain and two CBM6 domains, followed by a GH9 cellobiohydrolase (ID: 45317) and two GH10 xylanases (ID: 46613 and 44363). GH9 cellobiohydrolases catalyze the hydrolysis of crystalline regions, which is a critical step in cellulose degradation. A GH94 protein (ID: 44731) was also abundant on day 3 and showed 74% identity to a cellobiose phosphorylase from *Ruminiclostridium thermocellum*, which catalyzed the conversion of cellobiose to glucose and glucose-1-phosphate. Few enzymes known to be involved in lignin degradation were detected. However, an AA2 catalase/peroxidase affiliated with *Escherichia coli* was one of the highly expressed proteins.

By day 7, most of the above GH proteins exhibited increased abundances, although to different extents. Four xylanases, a GH11 xylanase from *Clostridium* (ID: 41662), a GH10 xylanase from *Lachnoclostridium* (ID: 46613), a GH10 xylanase from *Cellulosilyticum* (ID: 44363) and a GH10 xylanase from *Butyrivibrio* (ID: 42825) were among the five most overrepresented glycoside hydrolases. This result was in agreement with the high levels of xylanase activity in the supernatant and supported the fact that xylan was the main polysaccharide degraded and utilized by EMSD5. In contrast, only one cellulase, a GH9 endoglucanase from *Cellulosilyticum* (ID: 42825), was among the top five most abundant proteins, consistent with the comparatively low level of endoglucanase activity in the supernatant. During growth on corn stover, two multi-modular xylanases (ID: 41662 and 46613) had 88-fold and 42-fold increase in abundance, respectively. The high protein abundances of these two xylanases implied the pivotal role of these enzymes in the breakdown of xylan component of corn stover. A total of 11 GHs (1.3% of all proteins detected) involved in biomass degradation were identified across all three time points, including one α-amylase, one endoglucanase, one cellobiohydrolase, one β-glucosidase, four xylanases and one α-l-arabinofuranosidase. Based on protein abundance, these enzymes might provide the bulk of activities required for the decomposition of corn stover.

The quantitative proteomic data reported here showed an increase in the protein abundance of a core set of glycoside hydrolases over the cultivation period, including one endoglucanase (ID: 42825), one cellobiohydrolase (ID: 45317) and three xylanases (ID: 44819, 46613 and 41662). This could be due either to continuous expression or to release from the hydrolyzed substrate. However, the differential expression levels of these GHs at different stages were also observed, which may reflect the unique strategies employed by EMSD5 for degrading lignocellulose. At initial and middle stages of decomposition, EMSD5 mainly secreted multiple xylanases, accounting for xylan removal prior to cellulose degradation. Meanwhile, crystalline cellulose microfibrils were cleaved by cellobiohydrolases. At a later stage, endoglucanases appeared in high abundance, acting on the interior cellulose main chains exposed after hemicellulose removal. These findings suggested that the expression of glycoside hydrolases in EMSD5 were specifically regulated to efficiently break down the carbohydrate polymers in response to the ultrastructure of lignocellulosic matrix. This was in contrast to the multifunctional enzyme systems of *Caldicellulosiruptor*, in which constitutive expression of glycoside hydrolases and their induction by mono- and oligosaccharides have been observed [52].

Although the microbial consortium secreted multiple xylanases and cellulases, proteins in these sets differed substantially in abundance. For instance, the most highly expressed GH11 xylanase 41662 on day 7 was almost 40-fold more abundant than another identified GH11 xylanase 46506. While GH10 and GH11 xylanases were both involved in the hydrolysis of xylan main chains, enzymes assigned to these families were different in their substrate specificities. GH10 xylanases exhibited broad catalytic specificity and were capable of cleaving the glycosidic bonds next to substituted xylose residues [53]. In contrast, GH11 xylanases were tuned to act on unsubstituted xylan backbones [54]. Community members secreted a higher number of GH10 xylanases in high abundance probably because they were more active on highly branched xylanolytic substrates. There were two cellobiohydrolases, GH9 and GH48, identified in the corn stover-induced metaproteome. The GH9 cellobiohydrolase contained three CBM4 domains, a CBM30 domain and a CBM3 domain, while GH48 had no appended CBMs. It has been demonstrated that CBMs potentiate the capacities of cognate catalytic domains to degrade the target polysaccharides in intact plant cell walls through targeting and proximity effects [55]. One might infer that the GH9 cellobiohydrolase, with multiple CBMs, was induced earlier and to a higher level because it played a more important role in targeting and degrading the relatively intact plant cell walls at the initial stage.

Of the above quantified hydrolases, two GH3 β-glucosidases (ID: 45568 and 1391) showed relatively low abundances. Despite their detection in the supernatant, these cellobiose-hydrolyzing enzymes had no identifiable secretory signal peptides, raising the possibility that they were actually intracellularly located. As a matter of fact, the metagenome analysis identified 22 putative GH3 β-glucosidases and none of these enzymes were predicted to be secreted. The fact that we did not identify extracellular β-glucosidases was consistent with the absence of detectable glucose in the culture supernatant. This finding suggested that the cellulolytic apparatus of EMSD5 comprised two complementary components: the extracellular enzymes, including multi-modular endoglucanases and cellobiohydrolases, were responsible for the initial breakdown of insoluble cellulose to cello-oligosaccharides, while further hydrolysis of cellobiose took place mainly inside the cell by intracellular β-glucosidases. In support of this hypothesis, elevated expression levels of the above two β-glucosidases were observed during the course of cultivation on corn stover. Besides, a cytoplasmic GH94 cellobiose phosphorylase (ID: 44731) converting cellobiose to glucose and glucose-1-phosphate was detected in much higher abundance than these β-glucosidases. This result refined the model of intracellular cellobiose degradation. Thus, the consortium degraded cellobiose inside the cell through hydrolysis by GH3 β-glucosidases and/or through phosphorolysis by GH94 cellobiose phosphorylases. Cellulolytic filamentous fungi, by contrast, produced extracellular enzyme systems, including β-glucosidases, to completely break down cellulose to monosaccharides.

### Proteins with significant abundance changes over time

**Fig. 8:**
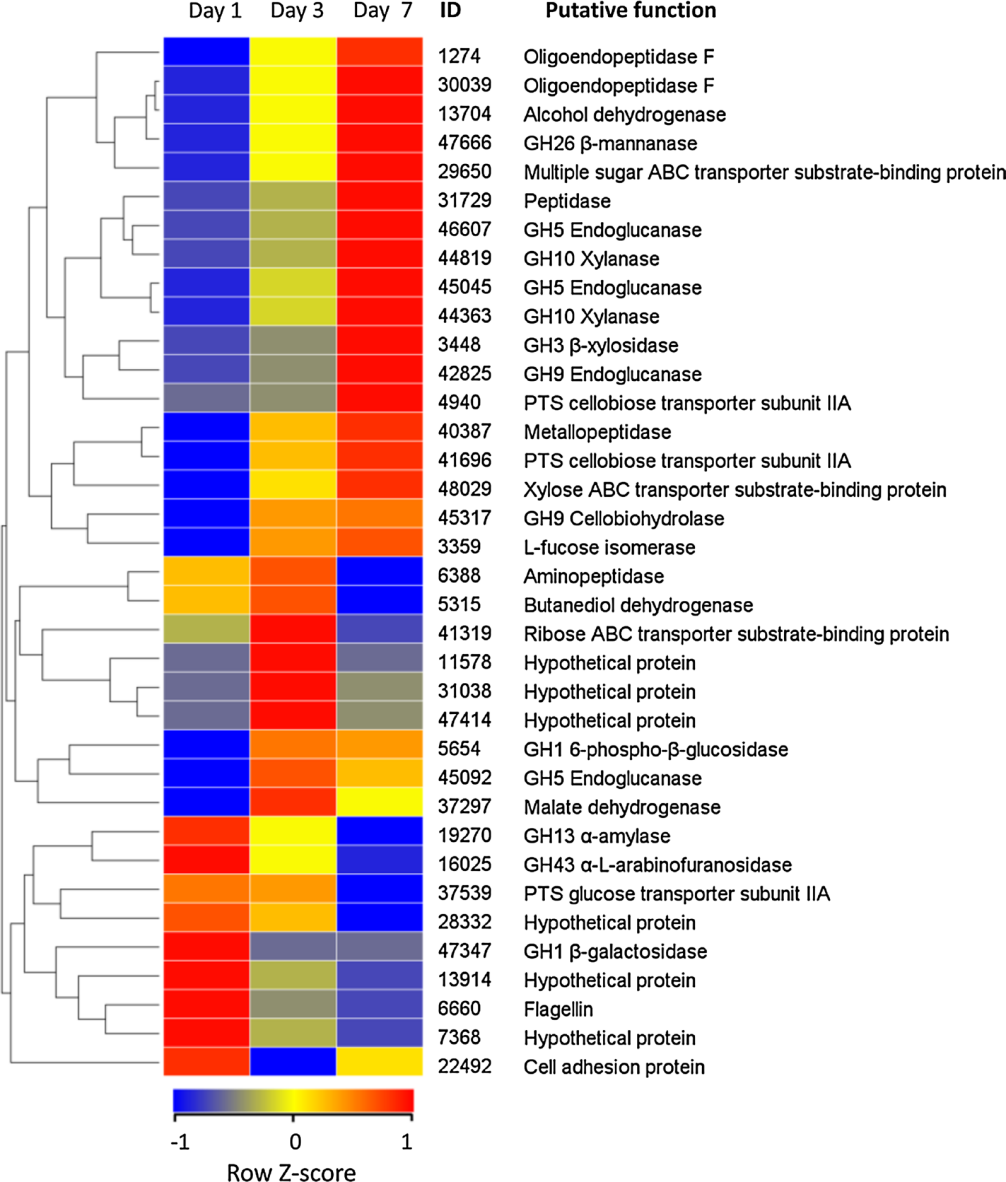
Hierarchical clustering of proteins with significant abundance change over time. The LFQ intensities of each protein across three time points are normalized by Z score transformation. Proteins exhibiting similar trend profiles were clustered into groups

To further interrogate the temporally differential expression patterns of extracellular proteins, the LFQ intensities of each protein were compared across three time points to identify proteins exhibiting significant abundance changes over time. A total of 36 proteins (14.1%) were found and categorized into groups based on abundance trend similarity. The abundances of eight glycoside hydrolases increased significantly during growth on corn stover (Fig. 8). This class included two xylanases, one β-xylosidase, one β-mannanase, three endoglucanases and one cellobiohydrolase. One of the endoglucanases, 42825, had an increase of over 300-fold in abundance. Multiple peptidases also followed this pattern. In addition, two PTS system cellobiose-specific subunits (ID: 4940 and 41496) were found to have significantly increased protein abundances over time. In bacteria the phosphotransferase system (PTS) participated in the uptake and phosphorylation of specific hexoses and disaccharides. As in our case, after being imported into the cell, phosphorylated cellobiose can be further hydrolyzed to glucose by intracellular GH1 6-phospho-β-glucosidases. This finding, together with the increased abundance of 6-phospho-β-glucosidase (ID: 16979), suggested a complementary pathway for processing cellobiose, which was different from the one involving intracellular β-glucosidases and cellobiose phosphorylases. Another hemicellulolytic enzyme with substantially increased abundance was the GH26 β-mannanase (ID: 47666). A multiple sugar ABC transporter substrate-binding protein (SBPs) (ID: 29650) was shown to have increased expression pattern similar to that of 47666, suggesting that it most likely had a substrate specificity for manno-oligosaccharides released by β-mannanases. Coordinate expression was also seen in xylanolytic enzymes and their associated substrate-binding proteins (ID: 48029), which were part of xylose-specific ABC transporter for the uptake of xylose released by these enzymes extracellularly. The significant increase in the protein abundance of 48029 was consistent with the high level of xylanase activity observed in the supernatant. Therefore, these SBPs could work in synergy with the major glycoside hydrolases of EMSD5.

Proteins declining significantly in abundance included a GH13 α-amylase (ID: 19270), a GH43 α-l-arabinofuranosidase (ID: 16025) and a GH1 β-galactosidase (ID: 47347). Three hypothetical proteins (ID: 28332, 13914 and 7368) clustered with these three GHs, showing a similar abundance pattern. While these previously uncharacterized proteins had no recognizable CAZy domains, their concurrent expression with these glycoside hydrolases suggested an unexplored role in polysaccharide deconstruction by the consortium. A PTS glucose transporter subunit (ID: 37539) showed decreased expression levels. This result was in accordance with the substantially low level of glucose in the culture supernatant over the cultivation period.

### Synergistic hydrolysis of pretreated lignocellulosic biomass

The metagenome and metaproteome analyses showed that the enzyme systems of EMSD5 grown on corn stover contained a wide diversity of hydrolytic enzymes, especially xylan-degrading enzymes. To evaluate the hydrolytic efficacy of EMSD5 enzymes acting on pretreated lignocellulosic materials, we performed hydrolysis experiments of delignified corn stover using enzyme preparation from EMSD5 cultivated on corn stover and a commercial enzyme preparation from *T. reesei*.

The two enzyme preparations showed significant differences in their specific activities against model cellulosic and xylanolytic substrates. The commercial preparation displayed higher levels of cellulase activities, including endoglucanase, cellobiohydrolase and β-glucosidase activities (Table 1). However, much higher levels of xylanase and β-xylosidase activities were present in the EMSD5 preparation, indicating its high hydrolytic activity toward xylan.

**Table 1:** Cellulase and hemicellulase activities of EMSD5 and commercial enzyme preparations

Enzyme source	Protein content (mg/ml)	CMCase (U/ml)	CBH (U/ml)	β-glucosidase (U/ml)	Xylanase (U/ml)	β-xylosidase (U/ml)
EMSD5	3.2 ± 0.2	11.1 ± 0.6	8.3 ± 0.2	4.4 ± 0.3	91.2 ± 4.3	17.0 ± 0.4
*T. reesei*	1.9 ± 0.1	32.7 ± 1.3	11.6 ± 0.4	20.9 ± 1.5	6.4 ± 1.8	3.2 ± 0.1

*CMCase* carboxymethyl cellulase,* CBH* cellobiohydrolase

When used individually, the two enzyme preparations exhibited remarkable differences in cellulose and xylan hydrolysis at the same protein loading. Enzyme preparation from the fungus *T. reesei* was more active on cellulose, while enzymes secreted by the bacterial consortium EMSD5 were more efficient in saccharifying the xylan component (Table 2). After 24 h of hydrolysis at an enzyme loading of 10 mg protein/g substrate, the commercial preparation alone converted 27.1% of cellulose in the pretreated corn stover and a much lower extent of xylan (2.2%). In contrast, the EMSD5 enzymes achieved a xylose yield of 18.7%, along with 8.1% cellulose conversion. We observed considerable synergistic enzyme activities on cellulose displayed by the combination of the two enzyme systems. Clearly, the addition of EMSD5 enzyme mixture to the commercial preparation, either as supplement or as replacement, resulted in improvements in the overall hydrolysis extent of pretreated corn stover. Supplementing commercial enzymes with increasing dosage of EMSD5 enzymes increased the cellulose and xylan conversions, while the degree of synergism remained around 1.2. Increased degree of synergism was observed when a higher proportion of fungal enzymes were replaced by bacterial enzymes. The highest degree of synergism (1.96) was obtained when up to 75% of fungal enzymes were replaced.

**Table 2:** Hydrolysis of delignified corn stover by commercial and EMSD5 enzyme preparations

Enzyme addition	Enzyme mixture	Protein loading (mg/g substrate)	Cellulose conversion (%)	Xylan conversion (%)	Degree of synergism
Individual	10 mg C	10	27.1 ± 0.6	2.2 ± 0.3	n.a.
	9 mg C	9	25.5 ± 0.3	2.1 ± 0.1	n.a.
	7.5 mg C	7.5	20.9 ± 0.3	1.9 ± 0.1	n.a.
	5 mg C	5	14.7 ± 0.2	1.4 ± 0.2	n.a.
	2.5 mg C	2.5	12.1 ± 0.4	1.0 ± 0.1	n.a.
	10 mg E	10	8.1 ± 0.2	18.7 ± 0.4	n.a.
	7.5 mg E	7.5	7.4 ± 0.3	16.3 ± 0.2	n.a.
	5 mg E	5	6.3 ± 0.1	12.2 ± 0.5	n.a.
	2.5 mg E	2.5	2.3 ± 0.1	5.6 ± 0.2	n.a.
	1 mg E	1	1.2 ± 0.3	2.7 ± 0.2	n.a.
Supplement	10 mg C + 2.5 mg E	12.5	34.7 ± 0.7	9.1 ± 0.5	1.18
	10 mg C + 5 mg E	15	40.5 ± 1.8	16.0 ± 0.5	1.21
	10 mg C + 7.5 mg E	17.5	43.1 ± 1.4	20.5 ± 0.8	1.25
	10 mg C + 10 mg E	20	44.6 ± 2.3	23.4 ± 0.6	1.27
Replace	9 mg C + 1 mg E	10	30.6 ± 0.9	4.9 ± 0.5	1.14
	7.5 mg C + 2.5 mg E	10	35.8 ± 0.6	8.8 ± 0.3	1.54
	5 mg C + 5 mg E	10	36.3 ± 1.7	15.1 ± 1.0	1.73
	2.5 mg C + 7.5 mg E	10	38.3 ± 1.3	18.7 ± 1.1	1.96

Hydrolysis assays were performed at 50 °C for 24 h at 2% (w/v) substrate loading

The mean values of three replicates and standard deviations are presented

*C* commercial enzyme preparation, *E* EMSD5 enzyme preparation. *n.a.* not applicable

From the above results, it was apparent that cellulase dosage was not the main factor influencing the effective hydrolysis of lignocellulosic biomass. The partial replacement of commercial cellulases with EMSD5 enzymes could considerably improve the hydrolysis yields of both cellulose and xylan present in pretreated corn stover, with significantly reduced cellulase protein loadings. A strong synergistic interaction between cellulases and xylanase was observed with respect to cellulose hydrolysis. During the saccharification of pretreated lignocellulosic biomass, access of cellulase enzymes to cellulose played a pivotal role in the efficiency of enzymatic hydrolysis [56]. In the delignified corn stover, considerable amount of hemicellulose, mostly xylan, remained associated with the cellulosic component. Cellulase enzymes alone had limited access to the cellulosic fiber surface due to the physical obstacles of the residual xylan. By partially replacing cellulase preparation with xylanase-enriched EMSD5 preparation, xylanases present in the resulting enzyme mixture enhanced the solubilization of xylan coating on the surface of cellulose fibers. The removal of xylan increased cellulose accessibility to the cellulase enzymes and, consequently, improved the effectiveness of enzyme hydrolysis of the cellulosic component. It has been suggested that the increasing cellulose accessibility by xylanase addition could also be a result of increasing fiber swelling and fiber porosity [57].

In the second-generation bioethanol industry, saccharification of cellulose and hemicellulose to fermentable sugars requires the use of multi-component enzyme mixtures. Most of the current commercial enzyme preparations were derived from filamentous fungi, such as *T. reesei* and *Aspergillus niger* [58]. The enzyme cocktails produced by these industrially relevant fungi comprise a large proportion of cellulases, whereas their hemicellulase component represents less than 1% of the total secreted proteins [59]. In this study, we demonstrated that supplementing fungal enzymes with hemicellulase-enriched mixture derived from bacterial consortia could enhance the release of glucose and xylose from pretreated lignocellulosic substrates. Moreover, moderate improvements in the polysaccharide conversions were observed when the commercial preparation was partially replaced by EMSD5 enzymes, without increasing the total enzyme loadings. The promoting effect of bacterial enzymes from EMSD5 on the hydrolysis effectiveness of fungal enzymes resulted mainly from the synergy between cellulases and hemicellulases. Supplementation or replacement of the commercial cellulases with xylanase-enriched preparations from a genetically modified strain of *T. reesei* has been demonstrated to increase the overall fermentable sugar yields from a range of pretreated lignocellulosic materials [57, 60]. Most research related to enzyme synergy has been focused on fungal enzymes [29, 57, 61], whereas synergistic interaction between fungal and bacterial enzymes has been studied to a much lesser extent. A broad range of hemicellulases from different bacterial sources have been demonstrated to synergize with fungal cellulases in the saccharification of ammonia fiber expansion-pretreated corn stover [62]. The enzyme mixture from the bacterial consortium EMSD5 was well suited for complementing fungal commercial preparations in the synergistic deconstruction of lignocellulosic materials, as it favored a diverse suite of accessory hemicellulolytic enzymes, such as xylanases, β-xylosidases, acetyl xylan esterases, α-l-arabinofuranosidases and α-glucuronidases.

## Conclusions

The present work demonstrated that the microbial consortium EMSD5 utilized a diverse complement of plant biomass-degrading bacteria and enzymes during growth on corn stover, especially those involved in hemicellulose decomposition. From the metagenomic analysis coupled with metaproteomic data, we revealed a central role of *Firmicutes* populations in biomass deconstruction and the synergistic cooperation of community members within the consortium. Furthermore, the quantitative proteome data in this study supported a model of sequential secretion of enzymes with different expression levels, consistent with the degradation of hemicellulose prior to cellulose by EMSD5. Our results also uncovered the potential roles of transport proteins and hypothetical proteins in the degradation of plant biomass. These findings not only expand the repertoire of microbial populations and enzymes implicated in lignocellulose deconstruction, but also more importantly advance our current understanding of microbial interaction and enzymatic synergism in this process. The enzymatic arsenal of EMSD5 provides a promising resource for mining efficient enzymes to improve the industrial enzymatic conversion process of lignocellulosic feedstock.

## Methods

### Cultivation of the microbial consortium

The microbial consortium EMSD5 was enriched from compost and preserved in 30% glycerol (v/v) at −70 °C. Prior to submerged fermentation, EMSD5 were incubated in 150 ml of seed medium (5.0 g/l tryptone; 5.0 g/l NaCl; 2.0 g/l CaCO_3_; 1.0 g/l yeast extract; 8.0 g/l corn stover, pH 7.0) at 37 °C for 5 days under static conditions for three consecutive transfers. Then, 1% of cultures of EMSD5 grown in seed medium (1 × 10^7^ cells/ml) were inoculated in triplicate in 500 ml Erlenmeyer flasks containing 300 ml of mineral salt medium (5.5 g/l NaCl; 2.5 g/l (NH_4_)_2_SO_4_; 0.1 g/l CaCl_2_·2H_2_O; 0.1 g/l MgSO_4_·7H_2_O; 5.3 g/l K_2_HPO_4_; 10.6 g/l KH_2_PO_4_, pH 7.0) supplemented with 3 g of corn stover, xylan or xylose. The cultures were cultivated under static conditions at 37 °C. The sugar contents of the culture supernatants, including glucose, xylose, cellobiose, xylobiose and arabinose, were analyzed by Essentia LC-15C high-performance liquid chromatography (Shimadzu, Japan) equipped with a Rezex ROA-Organic acid H+ (8%) column (Phenomenex, USA) and a RID-10A refractive index detector (Shimadzu, Japan). The following HPLC parameters were applied: an injection volume of 20 μl, 5 mM sulfuric acid as the mobile phase, a flow rate of 0.6 ml/min, and both column and detector temperatures set at 45 °C. The corn stover used in this study was composed of 39.1% cellulose, 17.6% xylan and 20.5% lignin. The structural carbohydrate and lignin contents of corn stover were determined according to the laboratory analytical procedure (LAP) of the National Renewable Energy Laboratory (NREL) (version 08-03-2012). In brief, 0.5 g of dry weight corn stover was subjected to sequential water and ethanol extraction at 95 °C. The extractives-free sample was hydrolyzed at 30 °C with 3.0 ml H_2_SO_4_ (72%) for 1 h. Then 84 mL of water was added and a second hydrolysis was carried out in the autoclave at 121 °C for 1 h. The mixture was then filtered by porcelain filter crucibles with glass filters. The glucose and xylose concentrations in the filtrates were determined by HPLC. The content of acid-insoluble lignin was determined by subtracting the ash content from the solid residue dried at 105 °C overnight. The ash content was determined by heating the solid residue at 575 °C for 3 h. The weight percentages of cellulose, xylan and lignin were calculated on an as-received biomass basis.

### DNA extraction and metagenome sequencing

After 5 days of cultivation in the corn stover-containing minimal medium, cultures were filtered through Miracloth (Merck) to remove solid residues. The cell pellets were collected by centrifugation at 6000*g* for 15 min at 4 °C. Total genomic DNA was extracted from the consortium with the TIANamp DNA Extraction Kit (TIANGEN, Beijing, China) according to the manufacturer’s instructions. The purity and quantity of the extracted DNA were analyzed using NanoDrop 2000 UV-Vis spectrophotometer (Thermo Scientific, USA) and Qubit 2.0 fluorometer (Life Technologies, USA). The DNA library with 300 bp insert size was constructed with Nextera DNA Library Preparation Kit (Illumina, USA) and sequenced on Illumina HiSeq 2500 platform. Raw reads from the metagenome sequencing were preprocessed using FASTX-Toolkit (version 0.0.14) with the following parameters: reads were removed if the percentage of low-quality bases (below quality 5) was above 40% of the read length or the percentage of N bases was above 10% of the read length, and adapters were removed (the overlapping length between reads and adapters was set at 15 bp). Duplicate reads were identified and removed using the CD-HIT program with default parameters [63]. After quality filtering and dereplication of the raw reads, de novo assembly was performed using the SOAPdenovo software (version 2.21) with a k-mer length of 43 [64]. The assembled contigs longer than 300 bp were subject to gene prediction using the MetaGeneMark software (version 2.10) with default parameters [65].

### Taxonomic and functional annotation of the metagenome data

Taxonomic annotation of open reading frames (ORFs) was performed by BLASTN search against the NCBI NT database, with an e-value cutoff of 1e-5. The blast hits were taxonomically assigned by the MEGAN software using the Lowest Common Ancestor (LCA) algorithm [66]. Functional annotation of predicted genes was performed by BLASTP search against the Kyoto Encyclopedia of Genes and Genomes (KEGG) database [67] and the evolutionary genealogy of genes: Non-supervised Orthologous Groups (eggNOG) database [68] using an e-value cutoff of 1e-5. Based on the relative abundance of the dominant bacterial phyla, metagenome data sets were highly consistent between replicates (Additional file 10: Figure S5).

### Carbohydrate-active enzyme annotation

Genes encoding carbohydrate-active enzymes were annotated using dbCAN based on the hidden Markov models (HMMs) of the signature domain of each CAZy family [69]. The following parameters were applied: coverage of at least 30% of the respective HMM, an e-value cutoff of 1e-5 for alignments longer than 80 aa and 1e-3 for alignments shorter than 80 aa. The predicted carbohydrate-active enzymes in the metagenome were additionally searched against the NCBI non-redundant database via PSI-BLASTP.

### Extracellular protein recovery

The supernatant fraction of cultures was collected by centrifugation at 10000*g* for 15 min at 4 °C and then filtered through an Acrodisc syringe filter with 0.20 μm Supor polyethersulfone (PES) membrane (PALL, USA). The filtrates were assayed directly for enzyme activities. For biomass saccharification assay, proteins in the supernatant were dialyzed against sodium acetate buffer (50 mM, pH 5.0) and then freeze-dried. For metaproteome analysis, soluble proteins in the filtered supernatant were precipitated with trichloroacetic acid (TCA). Briefly, ice-cold 100% TCA was added to a final concentration of 15% (v/v). The samples were incubated on ice for 20 min and subsequently overnight at −20 °C. Proteins were collected by centrifugation at 15,000*g* for 30 min at 4 °C. The protein pellets were washed three times with ice-cold 100% acetone, before air-drying for 5 min. The protein pellets were resuspended in 50 mM Tris-HCl with 150 mM NaCl (pH 8.0). The protein concentration was determined using the Bradford Protein Assay Kit (GenStar, China) according to the manufacturer’s instructions.

### Enzyme assays

The degradative enzyme activities in the culture supernatants were analyzed as previously described [70]. Briefly, activities of endoglucanase and xylanase were determined by the dinitrosalicyclic acid (DNS) method with low-viscosity carboxymethylcellulose (CMC) and beechwood xylan as substrates, respectively. 50 μl of culture filtrate was mixed with 150 μl of 1.0% (w/v) substrate in 50 mM sodium acetate buffer (pH 5.0) and incubated at 50 °C for 10 min. The reaction was terminated by adding 50 μl of 1 M NaOH. After boiling at 100 °C for 5 min, the concentrations of reducing sugar were measured at 540 nm. Activities were calculated with glucose or xylose as the standard. One unit of enzyme activity was defined as the amount of enzyme catalyzing the release of 1 μmol of reducing sugars in 1 min from the substrate under the above conditions.

The activities of cellobiohydrolase, β-glucosidase, β-xylosidase, α-l-arabinofuranosidase and xylan esterase were assayed using the respective substrates* p*-nitrophenyl-β-d-cellobioside (pNPC),* p*-nitrophenyl-β-d-glucopyranoside (pNPG),* p*-nitrophenyl-β-d-xylopyranoside (pNPX),* p*-nitrophenyl-α-l-arabinofuranoside (pNPAF) and p-nitrophenyl-acetate (pNPAC). The reaction mixture contained 50 μl of culture filtrate, 50 μl of 200 mM sodium acetate buffer (pH 5.0) and 100 μl of 5 mM substrate solution. After incubation at 50 °C for 10 min, the reaction was terminated by adding 100 μl 1 M Na_2_CO_3_ and the absorbance of released p-nitrophenol was measured at 405 nm. Activities were calculated using p-nitrophenol as the standard. One unit of enzyme activity was defined as the amount of enzyme that produced 1 μmol of pNP in 1 min from the substrate under the above conditions.

### Enzymatic hydrolysis of pretreated corn stover

Corn stover was partially delignified with sodium chlorite before hydrolysis according to the procedure in the Pulp and Paper Technical Association of Canada’s (PAPTAC) Useful methods G10.U. Pretreated corn stover was composed of 46.0% cellulose, 20.4% xylan and 9.9% lignin. The hydrolysis assay was carried out at 2% (w/v) substrate loading in 1 ml of total volume. The commercial enzyme preparation used in this study was derived from *Trichoderma reesei* ATCC 26921 (C8546, Sigma). The enzyme preparation derived from EMSD5 was added to the commercial preparation in two different ways: supplementation and replacement. In the supplementation approach, increasing amounts of EMSD5 enzymes (2.5-10 mg/g substrate) were added to the commercial enzymes (10 mg/g substrate). In the replacement approach, the commercial preparation was replaced in increasing proportion (up to 75% based on mass) with an equal amount of EMSD5 enzymes, while the total enzyme loading was kept constant at 10 mg/g substrate. Enzyme-substrate mixture in sodium acetate buffer (50 mM, pH 5.0) was incubated at 50 °C in an orbital shaker incubator. To terminate the hydrolysis, the reaction mixture was incubated at 100 °C for 10 min. The hydrolysates were collected by centrifugation at 14,000*g* for 10 min and then filtered through a 0.45 μm filter. The concentrations of glucose and xylose in the filtrates were determined by HPLC. The cellulose and xylan conversions were calculated based on the initial contents in the pretreated corn stover. The degree of synergism was calculated as the ratio of the sum of cellulose conversion achieved with each enzyme in individual hydrolysis and the cellulose conversion achieved with the combination of all enzymes in one hydrolysis reaction. All hydrolysis experiments were conducted in triplicate and mean values and standard deviations are presented.

### NanoLC-MS/MS analysis of extracellular metaproteome

For each biological replicate, 25 μg of protein was separated by SDS-PAGE using 12.5% polyacrylamide at 100 V. Each lane was divided into six fractions. The gel pieces were subject to tryptic digestion as previously described [70]. The resulting peptides were reconstituted in 0.1% formic acid before nanoLC-MS/MS.

Separation of peptide mixture was performed on a nanoAcquity UPLC (Waters, Milford, MA, USA). The trap column was made with 100 μm I.D. fused silica capillary (Polymicro, Phoenix, AZ, USA) filled with 20 mm of C18 stationary phase (Phenomenex, Torrance, CA, USA). The analytical column was made with 50 μm I.D. fused silica capillary (Polymicro) filled with 10 cm of C18 stationary phase. Peptides were loaded onto the trap column and eluted using a 100 min gradient from 99% mobile phase A (0.1% formic acid in water) to 40% mobile phase B (0.1% formic acid in acetonitrile) for 80 min, 40–80% mobile phase B for 10 min and 80% mobile phase B for 10 min, at a flow rate of 200 nl/min. The subsequent nanospray ESI-MS was performed on a Q-Exactive high-resolution mass spectrometer (Thermo Scientific, Waltham, MA, USA). The mass spectrometer was set in a data-dependent MS2 mode. Full-scan MS ranged from m/z 300 to 2000 with a resolution of 70,000. The ten most abundant ions from the full scan were selected for MS/MS scans.

For protein identification, raw data from MS were preprocessed with Mascot Distiller 2.5 for peak picking. The resulting peaks were searched against a database containing all predicted protein sequences from EMSD5 metagenome using Mascot search engine (version 2.5.1). The following parameters were applied: carbamidomethyl cysteine as fixed modification and oxidized methionine as the variable modification. A maximum of two missed tryptic cleavages were allowed. The peptide mass tolerance was set to 15 ppm and MS/MS fragment mass tolerance was set to 0.02 Da. Protein false discovery rate (FDR) was adjusted to 1%. A minimum of two significant peptides and one unique peptide were required for each identified protein. The theoretical isoelectric point (pI) and molecular weight (Mw) of identified proteins were calculated using the Compute pI/Mw tool in ExPASy (http://web.expasy.org/compute_pi). Secretion signals in the identified proteins were predicted by SignalP 4.1 server (http://www.cbs.dtu.dk/services/SignalP/). A supplementary figure was provided to illustrate the reproducibility between metaproteome replicates (Additional file 11: Figure S6).

### Label-free quantification of time-course extracellular metaproteomes

MaxQuant (version 1.4.1.2) [71] with Andromeda [72] as the search engine was used for identification and quantification of extracellular proteins on days 1, 3 and 7. Two technical replicates were performed for each of the three biological replicates. MS spectra were searched against the same protein database derived from EMSD5 metagenome with revert decoy mode. Trypsin was used as the enzyme and up to two missed cleavages were allowed. Carbamidomethylation of cysteines was set as a fixed modification, and acetylation of protein N-terminus and oxidation of methionines were set as variable modifications. The MS/MS tolerance was set to 20 ppm, and fragment mass tolerance was set to 0.5 Da. Protein and peptide false discovery rate (FDR) were adjusted to 1%. ‘Match between runs’ option was enabled with 1 min match time window and 20 min alignment time window. A minimum of two ratio counts were required for valid protein quantification. The remaining parameters were kept as default. ‘Fast LFQ’ option was disabled and ‘intensity-based absolute quantification (iBAQ)’ option was enabled. Only proteins identified with at least two peptides were considered for label-free quantification. Calculation of the protein LFQ intensity was based on unique peptides using the built-in label-free quantification algorithm [73]. Proteins showing significant differences in abundance over time (P < 0.05) were determined by one-way analysis of variance (ANOVA) using SPSS Statistics 19. Replicate-to-replicate variation was assessed by Pearson correlation analysis using all LFQ intensities (Additional file 12: Table S6).

## Additional files

**Additional file 1: Figure S1.** Figure S1.pdf. Xylanase and endoglucanase activities of culture supernatants produced by EMSD5 cultivated on corn stover. The values shown are the mean of three replicates and the error bars indicate standard deviations from the mean values.

**Additional file 2: Table S1.** Table S1.docx. de novo assembly results of EMSD5 metagenome.

**Additional file 3: Figure S2.** Figure S2.pdf. Functional classification of predicted proteins in the metagenome based on **a** the KEGG orthology system and **b** the EggNOG database.

**Additional file 4: Table S2.** Table S2.xlsx. Distribution of CAZy family proteins in the metagenome of EMSD5. CAZy family of predicted proteins was analyzed with dbCAN.

**Additional file 5: Figure S3.** Figure S3.pdf. **a** Number of CAZyme genes in the genomes of 14 genera and **b** Taxonomic distribution of potential carbohydrate-active enzymes in the metagenome.

**Additional file 6: Table S3.** Table S3.xlsx. Proteins identified in the culture supernatants of corn stover-, xylan- and xylose-containing medium on day 5, respectively.

**Additional file 7: Table S4.** Table S4.docx Plant biomass-degrading proteins detected in corn stover-induced metaproteome after 5 days of cultivation.

**Additional file 8: Figure S4.** Figure S4.pdf. Comparison of cellulase and hemicellulase activities of the extracellular proteins produced by EMSD5 cultivated on three carbon sources. The values shown are the mean of three replicates and the error bars indicate standard deviations from the mean values.

**Additional file 9: Table S5.** Table S5.xlsx. Abundance profiles of all identified proteins across three time points on corn stover.

**Additional file 10: Figure S5.** Figure S5.pdf. Relative abundance of dominant bacterial phyla in the metagenome replicates.

**Additional file 11: Figure S6.** Figure S6.pdf. Venn diagrams of proteins identified in the replicates of each substrate. The substrates used in this study include **a** corn stover, **b** xylan and **c** xylose.

**Additional file 12: Table S6.** Table S6.docx. Protein abundance correlations showing reproducibility of label-free quantification between the replicates. The values shown are the Pearson correlation coefficients.

## Abbreviations

CMCase: carboxymethyl cellulase
FPA: filter paper activity
CBH: cellobiohydrolase
GH: glycoside hydrolase
CBM: carbohydrate-binding module
NanoLC–MS/MS: nano liquid chromatography-tandem mass spectrometry
ORF: open reading frame
CAZyme: carbohydrate-active enzyme
GT: glycosyltransferase
CE: carbohydrate esterase
AA: auxiliary activities
PL: polysaccharide lyase
DNS: 3,5-dinitrosalicylic acid
CMC: carboxymethyl cellulose
pNPC: p-nitrophenyl-β-d-cellobioside
pNPG: p-nitrophenyl-β-d-glucopyranoside
pNPX: p-nitrophenyl-β-d-xylopyranoside
pNPAF: p-nitrophenyl-α-l-arabinofuranoside
pNPAC: p-nitrophenyl-acetate
KEGG: Kyoto Encyclopedia of Genes and Genomes
eggNOG: evolutionary genealogy of genes: Non-supervised Orthologous Groups
COGs: clusters of orthologous groups
PSI-BLAST: position-specific iterated-BLAST
HMM: hidden Markov model
TCA: trichloroacetic acid
