# Supplementary figures and images for: Metagenomic and metaproteomic analyses of a corn stover-adapted microbial consortium EMSD5 reveal its taxonomic and enzymatic basis for degrading lignocellulose
Source: Biotechnol Biofuels. 2016 Nov 9;9:243. doi: 10.1186/s13068-016-0658-z (PMC5103373; doi:10.1186/s13068-016-0658-z)

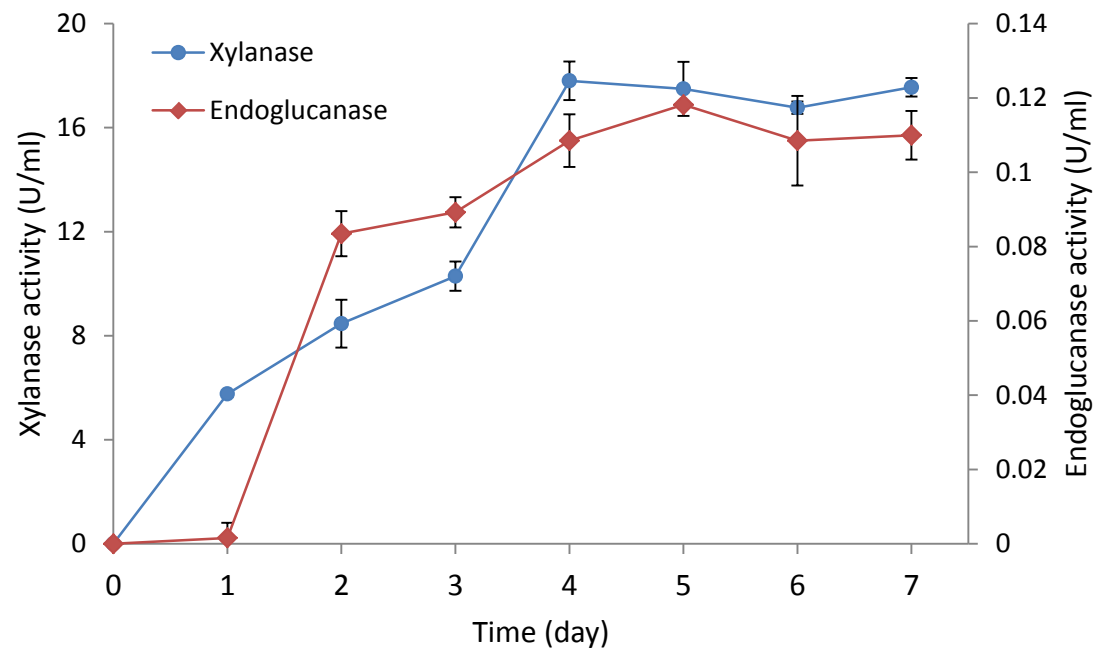

Supplement: Supplementary file 1 — Additional file 1: Figure S1. Figure S1.pdf. Xylanase and endoglucanase activities of culture supernatants produced by EMSD5 cultivated on corn stover. The values shown are the mean of three replicates and the error bars indicate standard deviations from the mean values. [file 13068_2016_658_MOESM1_ESM.pdf]

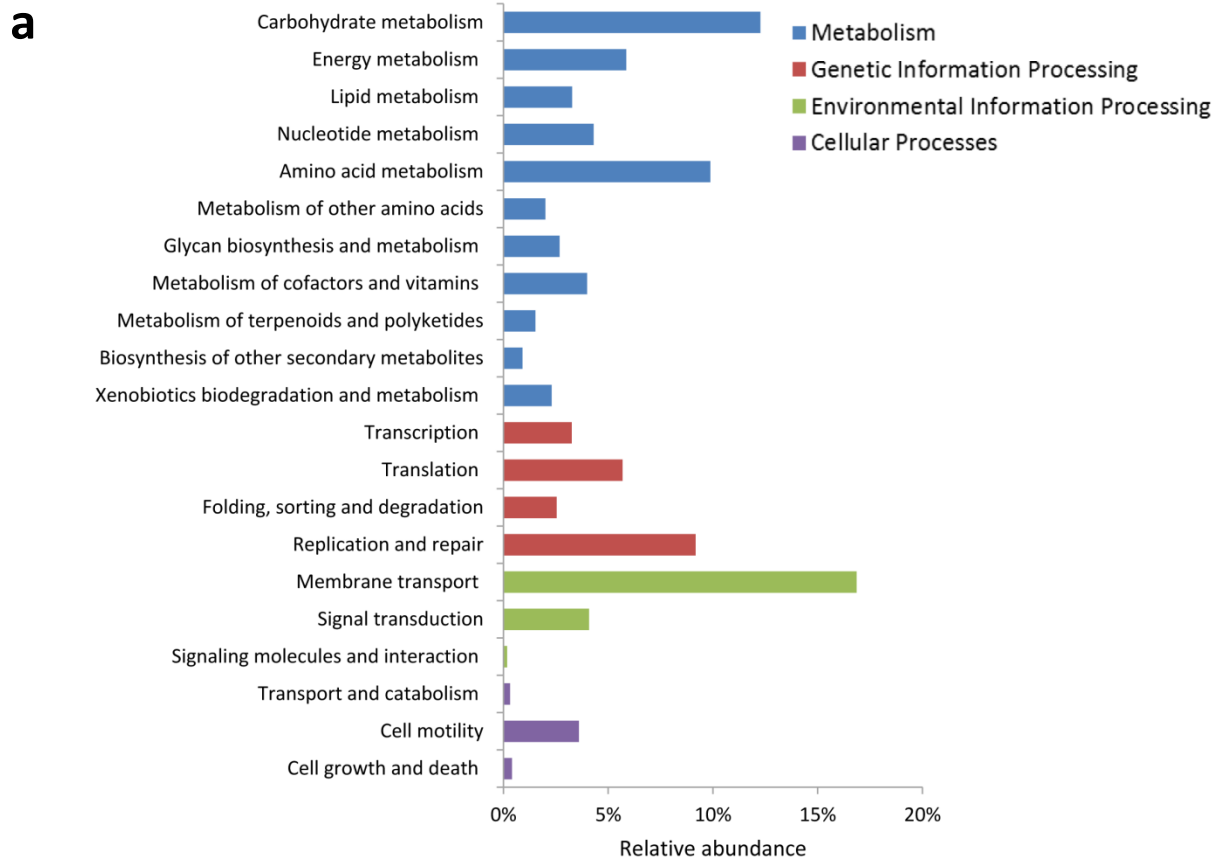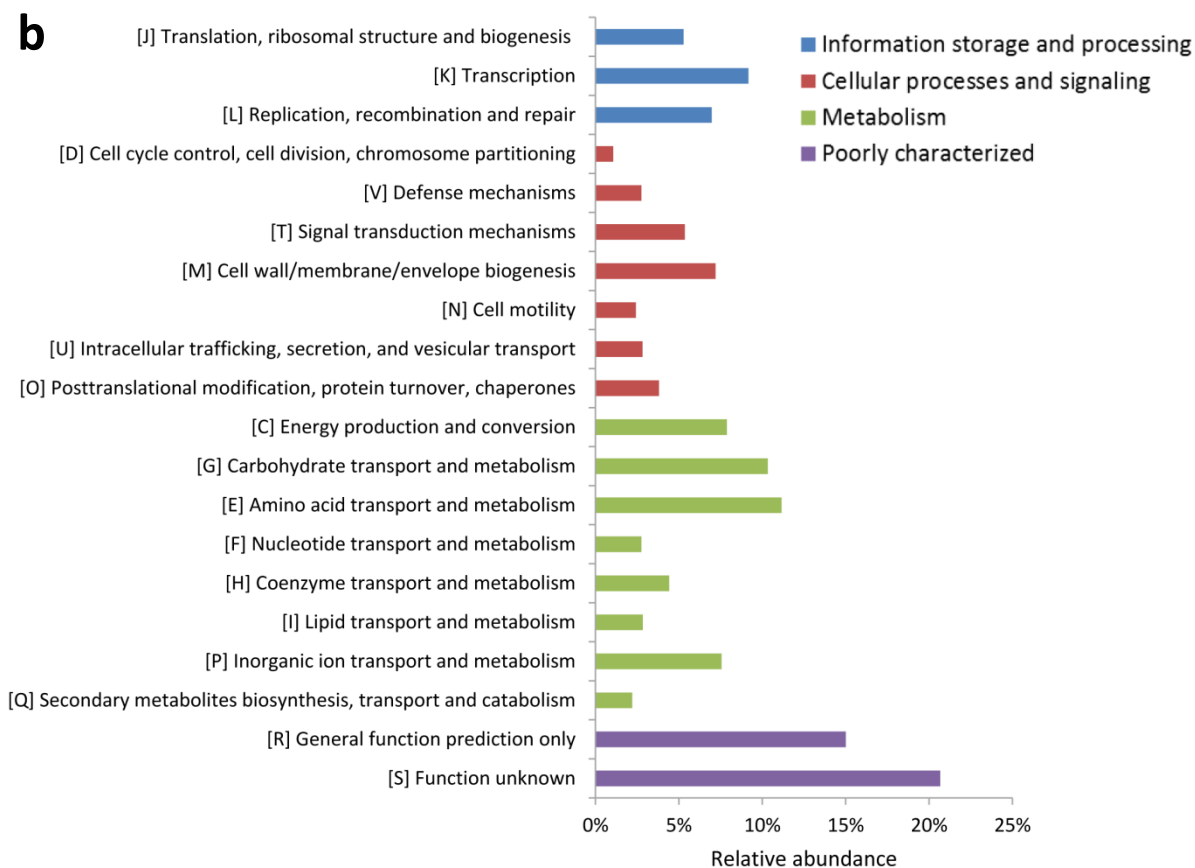

Supplement: Supplementary file 3 — Additional file 3: Figure S2. Figure S2.pdf. Functional classification of predicted proteins in the metagenome based on a the KEGG orthology system and b the EggNOG database. [file 13068_2016_658_MOESM3_ESM.pdf]

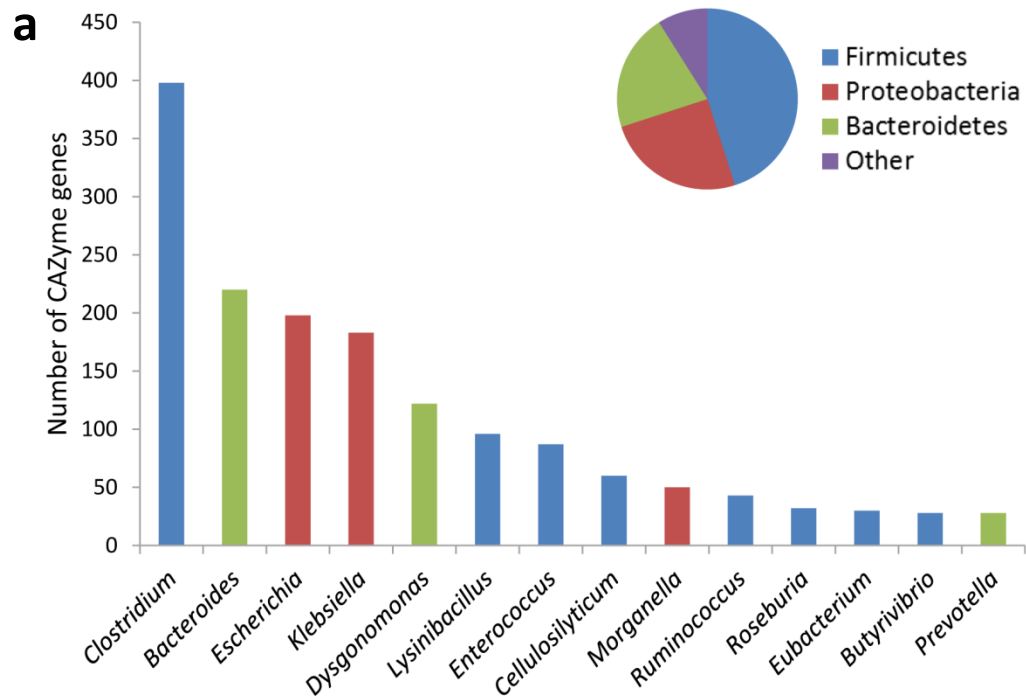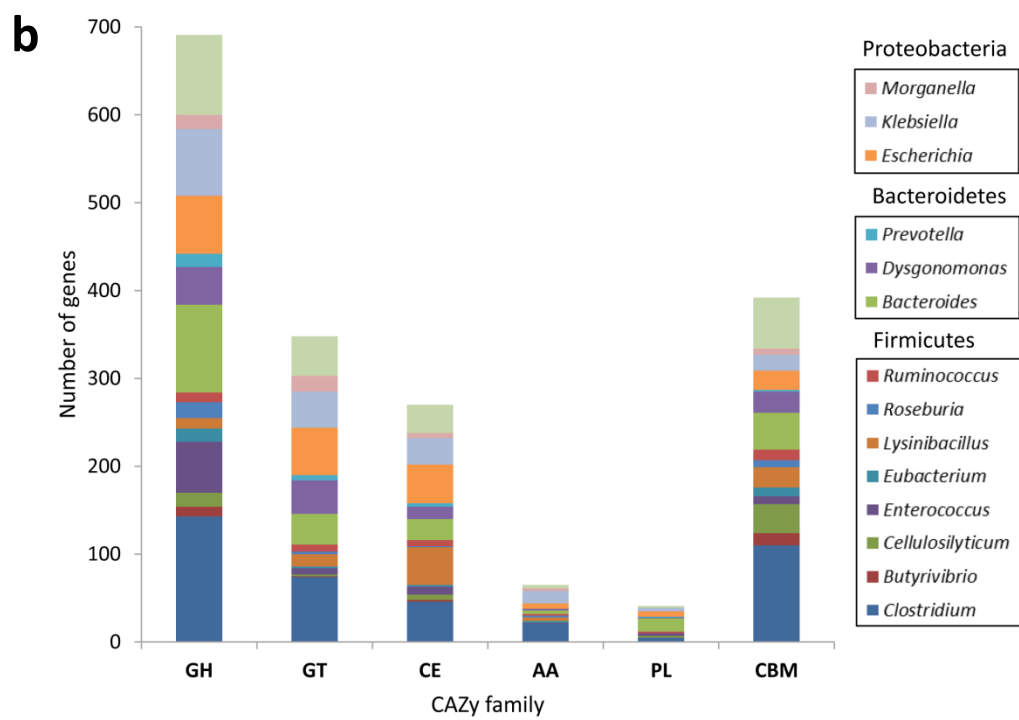

Supplement: Supplementary file 5 — Additional file 5: Figure S3. Figure S3.pdf. a Number of CAZyme genes in the genomes of 14 genera and b Taxonomic distribution of potential carbohydrate-active enzymes in the metagenome. [file 13068_2016_658_MOESM5_ESM.pdf]

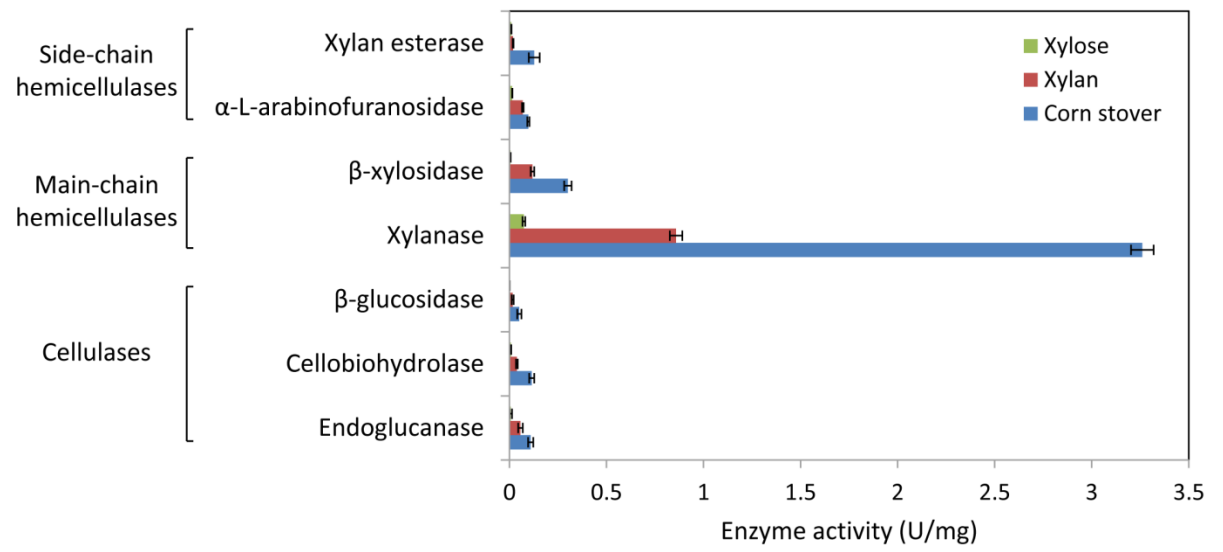

Supplement: Supplementary file 8 — Additional file 8: Figure S4. Figure S4.pdf. Comparison of cellulase and hemicellulase activities of the extracellular proteins produced by EMSD5 cultivated on three carbon sources. The values shown are the mean of three replicates and the error bars indicate standard deviations from the mean values. [file 13068_2016_658_MOESM8_ESM.pdf]

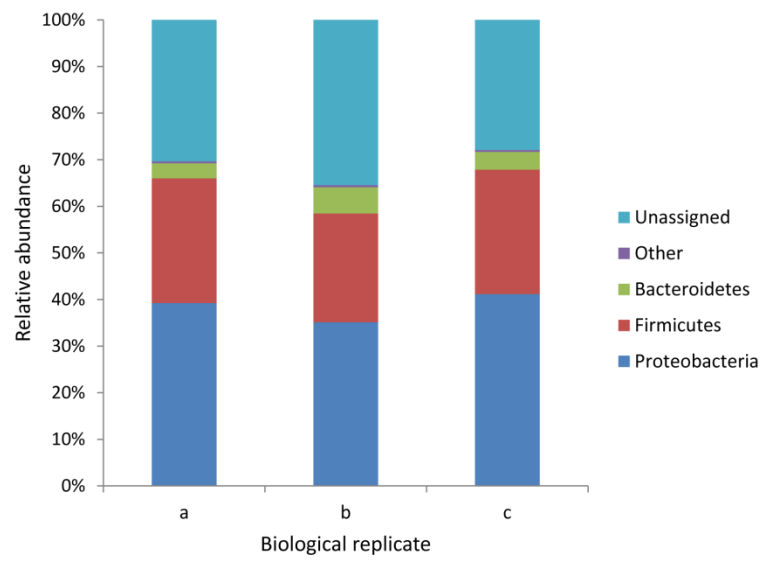

Supplement: Supplementary file 10 — Additional file 10: Figure S5. Figure S5.pdf. Relative abundance of dominant bacterial phyla in the metagenome replicates. [file 13068_2016_658_MOESM10_ESM.pdf]

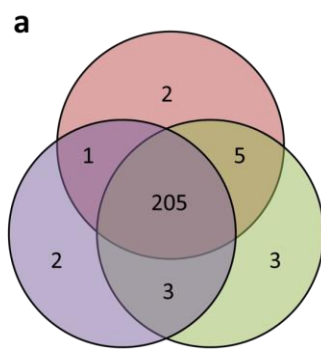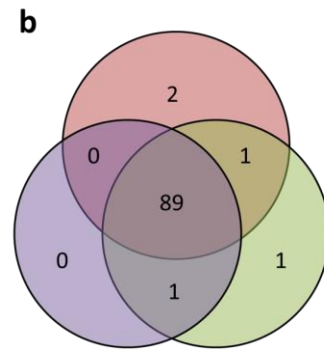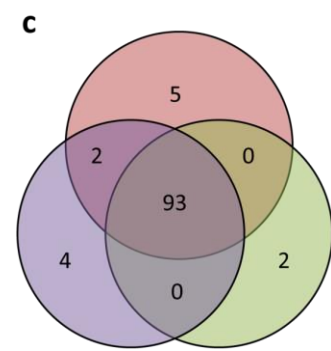

Supplement: Supplementary file 11 — Additional file 11: Figure S6. Figure S6.pdf. Venn diagrams of proteins identified in the replicates of each substrate. The substrates used in this study include a corn stover, b xylan and c xylose. [file 13068_2016_658_MOESM11_ESM.pdf]
