# Supplementary material for: Metagenomic and metaproteomic analyses of a corn stover-adapted microbial consortium EMSD5 reveal its taxonomic and enzymatic basis for degrading lignocellulose
Source: Biotechnol Biofuels. 2016 Nov 9;9:243. doi: 10.1186/s13068-016-0658-z (PMC5103373; doi:10.1186/s13068-016-0658-z)
Supplement: Supplementary file 2 — Additional file 2: Table S1. Table S1.docx. de novo assembly results of EMSD5 metagenome. [file 13068_2016_658_MOESM2_ESM.docx]

**Table S1 *de novo* assembly results of EMSD5 metagenome**

| ID | Clean reads | Contig number | Total length  (bp) | Avergage length  (bp) | N50  (bp) | N90  (bp) | Max length  (bp) |
| --- | --- | --- | --- | --- | --- | --- | --- |
| EMSD5 | 864,196 | 17,908 | 39,141,874 | 2185 | 16,757 | 640 | 365,763 |
