# Supplementary material for: Metagenomic and metaproteomic analyses of a corn stover-adapted microbial consortium EMSD5 reveal its taxonomic and enzymatic basis for degrading lignocellulose
Source: Biotechnol Biofuels. 2016 Nov 9;9:243. doi: 10.1186/s13068-016-0658-z (PMC5103373; doi:10.1186/s13068-016-0658-z)
Supplement: Supplementary file 7 — Additional file 7: Table S4. Table S4.docx Plant biomass-degrading proteins detected in corn stover-induced metaproteome after 5 days of cultivation. [file 13068_2016_658_MOESM7_ESM.docx]

**Table S4 Plant biomass-degrading proteins detected in corn stover-induced metaproteome after 5 days of cultivation**

| Substrate | ID | Domain organization | Organism source | SP | Blastp hit | Coverage | Identity |
| --- | --- | --- | --- | --- | --- | --- | --- |
| Cellulose | 45045 | CBM2-GH5 | *Cellulosilyticum* | Y | CDC66676.1 | 94% | 51% |
|  | 45092 | GH5-CBM2 | *Clostridium cellobioparum* | Y | CCZ55578.1 | 76% | 61% |
|  | 46607 | GH5-CBM46-CBM3 | *Eubacterium cellulosolvens* | Y | WP_051527159.1 | 58% | 45% |
|  | 42825 | GH9-CBM3-CBM3 | *Cellulosilyticum lentocellum* | Y | WP_013655743.1 | 99% | 46% |
|  | 45635 | GH9-CBM3-CBM3 | *Cellulosilyticum lentocellum* | Y | WP_013658626.1 | 92% | 57% |
|  | 45317 | CBM4-CBM4-CBM4-CBM30-GH9-CBM3 | *Clostridium saccharoperbutylacetonicum* | Y | ADZ82408.1 | 97% | 50% |
|  | 42775 | GH48 | *Clostridium cellulolyticum* | Y | WP_013658627.1 | 93% | 57% |
|  | 5654 | GH1 | *Clostridium butyricum* | N | WP_035763054.1 | 100% | 100% |
|  | 1391 | GH3 | *Bacteroides coprosuis* | N | WP_006744863.1 | 100% | 100% |
| Xylan (main chain) | 44819 | CBM22-CBM22-GH10-CBM3 | *Cellulosilyticum lentocellum* | Y | WP_013658377.1 | 77% | 52% |
|  | 44363 | CBM22-CBM22-GH10-CBM22-CBM22-CBM22 | *Roseburia intestinalis* | Y | CUN30395.1 | 56% | 46% |
|  | 41662 | GH11-CBM6-CBM6 | *Ruminococcus* | Y | WP_034836380.1 | 95% | 65% |
|  | 46506 | GH11-CBM13-CBM2 | *Cellulosilyticum ruminicola* | N | CDD34503.1 | 40% | 78% |
|  | 46613 | GH10-CBM3 | *Lachnoclostridium phytofermentans* |  | WP_012199538.1 | 71% | 56% |
|  | 48211 | GH10-CBM13-CE1-CBM2 | *Butyrivibrio fibrisolvens* | Y | WP_051212946.1 | 67% | 56% |
|  | 3448 | GH3 | *Flavobacterium johnsoniae* | N | CAD48309.1 | 97% | 63% |
|  | 45315 | GH39-CBM13-CBM13 | *Clostridium saccharoperbutylacetonicum* | Y | WP_009170329.1 | 56% | 68% |
|  | 44723 | GH43-CBM13-CBM2 | *Cellulosilyticum ruminicola* | Y | ACZ98626.1 | 73% | 55% |
|  | 28791 | GH43 | *Sphaerochaeta coccoides* | N | WP_013739293.1 | 99% | 64% |
| Xylan (side chain) | 30845 | GH43 | *Klebsiella pneumoniae* | N | WP_019705619.1 | 100% | 99% |
|  | 16025 | GH43 | *Enterococcus mundtii* | N | WP_016612864.1 | 100% | 99% |
|  | 16248 | GH51 | *Enterococcus casseliflavus* | N | WP_016611392.1 | 100% | 99% |
|  | 43736 | CBM9-CE1-CBM9-CE1 | *Clostridium clariflavum* | Y | WP_024832026.1 | 72% | 56% |
|  | 46503 | CBM36-CE4 | *Pseudobutyrivibrio xylanivorans* | N | P83513.2 | 99% | 65% |
|  | 15896 | CE4 | *Enterococcus casseliflavus* | N | WP_035006642.1 | 100% | 99% |
|  | 29651 | GH67 | *Paenibacillus* | N | WP_045085083.1 | 100% | 72% |
| Xyloglucan | 34002 | GH31 | *Paenibacillus mucilaginosus* | N | WP_045085863.1 | 99% | 70% |
| Mannan | 44736 | CBM27-CBM27-GH26-CBM23-CBM3 | *Roseburia intestinalis* | Y | CDF45187.1 | 85% | 46% |
|  | 47666 | CBM35-GH26-CBM3 | *Clostridium clariflavum* | Y | WP_007428240.1 | 91% | 68% |
|  | 46460 | GH5-CBM32-CBM46-CBM46-CBM46-CBM46-CBM2 | *Cellulosilyticum lentocellum* | Y | WP_013656979.1 | 95% | 51% |
| Pectin | 24823 | GH2 | *Clostridium* | N | WP_010073567.1 | 98% | 62% |
| Starch | 19270 | GH13-CBM26-CBM26-CBM26-CBM26-CBM26-CBM26 | *Clostridium saccharoperbutylacetonicum* | Y | WP_035763668.1 | 100% | 99% |
| (1,3;1,4)-β glucan | 42535 | GH16-CBM4-CBM4-CBM4-CBM4 | *Clostridium perfringens* | Y | WP_058372076.1 | 89% | 50% |
| Lignin | 40694 | AA2 | *Escherichia coli* | N | EHP63591.1 | 100% | 100% |
