# Supplementary material for: Metagenomic and metaproteomic analyses of a corn stover-adapted microbial consortium EMSD5 reveal its taxonomic and enzymatic basis for degrading lignocellulose
Source: Biotechnol Biofuels. 2016 Nov 9;9:243. doi: 10.1186/s13068-016-0658-z (PMC5103373; doi:10.1186/s13068-016-0658-z)
Supplement: Supplementary file 12 — Additional file 12: Table S6. Table S6.docx. Protein abundance correlations showing reproducibility of label-free quantification between the replicates. The values shown are the Pearson correlation coefficients. [file 13068_2016_658_MOESM12_ESM.docx]

**Table S6 Protein abundance correlations showing reproducibility of label-free quantification between the replicates.**

|  | Day 1-A | Day 1-B |  |  | Day 3-A | Day 3-B |  |  | Day 7-A | Day 7-B |  |
| --- | --- | --- | --- | --- | --- | --- | --- | --- | --- | --- | --- |
| Day 1-B | 0.979 |  |  | Day 3-B | 0.928 |  |  | Day 7-B | 0.862 |  |  |
| Day 1-C | 0.964 | 0.932 |  | Day 3-C | 0.935 | 0.893 |  | Day 7-C | 0.871 | 0.941 |  |

The values shown are the Pearson correlation coefficients.
